# Spontaneous Induced Cascade Targeting Biomimetic Nanoparticles to Inhibit Dendritic Cell Maturation for Ameliorating Atherosclerosis and Magnetic Resonance Imaging

**DOI:** 10.34133/bmr.0204

**Published:** 2025-05-09

**Authors:** Danyan Li, Pengzhao Chang, Shuang Bian, Bangbang Li, Yangang Zhu, Yanchen Wang, Pingfu Hou, Jingjing Li

**Affiliations:** ^1^ School of Medical Imaging, Xuzhou Medical University, Xuzhou 221004, China.; ^2^ Department of Radiology, Affiliated Hospital of Xuzhou Medical University, Xuzhou 221006, China.; ^3^ Cancer Institute, Xuzhou Medical University, Xuzhou, Jiangsu 221004, China.

## Abstract

The major fatal factor of cardiovascular disease is atherosclerosis, which is a chronic inflammatory disease featured by immune cell infiltration within arterial plaques. Dendritic cells (DCs) are central stimulators of atherosclerotic inflammation, with mature DCs generating pro-inflammatory signals within plaque lesions and tolerogenic DCs promoting anti-inflammatory cytokine production and regulatory T cell (T_reg_) activation. In this work, spontaneous induced cascade targeting biomimetic nanoparticles (MM@HGPBRD) were constructed to target DCs in atherosclerosis plaques to inhibit DC maturation. In vitro and in vivo experiment results showed that the MM@HGPBRD effectively slowed atherosclerosis progression by the synergistic effect of multiple components. The coating macrophage membrane helped the nanoparticles to evade immune clearance and home to the atherosclerotic site. Then, the nanozyme activity of hollow mesoporous Prussian blue (HGPB) produced oxygen to break the membrane and expose DC-SIGN aptamer to realize cascade targeting to DCs and enhance the targeted release of rapamycin (RAPA) to inhibit DC maturation. The whole process regulated the inflammatory and immune microenvironment of atherosclerosis. At the same time, the excellent magnetic resonance imaging (MRI) ability of HGPB favored the MRI of DCs in atherosclerosis plaque. This study provides new avenue for spontaneous induced cascade targeting and modulating DC maturation to improve atherosclerosis inflammation and immune microenvironment.

## Introduction

Atherosclerosis is a chronic inflammatory and autoimmune disorder that is the leading cause of acute cardiovascular and a serious danger to the life and health of patients [1]. Previously, it was thought that atherosclerosis was caused by lipid deposition in the arterial wall. Based on this mechanism, clinical treatment of atherosclerosis relies mainly on lipid-lowering drugs, especially statins [2,3]. However, the drawbacks of poor solubility of free drug, rapid drug clearance, and low bioavailability have limited the effectiveness of clinical treatment of atherosclerosis [4].

Immunomodulatory therapy has become a powerful tool for clinical treatment. It has been extensively applied in the therapy of numerous illnesses, especially cancer [5]. Notably, there is a growing evidence that a good deal of immune cells are relevant to atherosclerotic plaque development and progression [6]. Among them, macrophages [7], neutrophils [8], DC [9,10], natural killer (NK) cells, and T lymphocytes are common [11,12]. The combination of these cells and the various cytokines they secrete constitute the atherosclerotic immune microenvironment [13]. The current research on immunotherapy for atherosclerosis mainly focuses on the macrophage immunotherapy. For example, Zhou et al. [14] remodeled the atherosclerotic immune microenvironment by blocking macrophage lipid antigen presentation to adaptive immune cells to inhibit the advancement of atherosclerosis. Gao et al. [15] developed a macrophage mRNA-targeted nanotherapeutic approach that could selectively deliver interleukin-10 (IL-10) mRNA to the plaque inflammatory milieu and promote macrophage polarization toward an M2 anti-inflammatory phenotype. Such role could create a beneficial positive feedback circulation to improve the inflammatory microenvironment. He et al. [16] utilized resolvin D1-loaded 2-dimensional black phosphorus nanosheets to selectively deliver resolvin D1 into diseased macrophages and scavenge reactive oxygen species (ROS) to realize anti-atherosclerotic efficacy.

In recent years, DCs have been found to be the best-performing antigen-presenting cells in the immunological system, serving as an intermediate node between the innate and adaptive immune systems. This distinctive location enables DCs to be an essential target for immunotherapy of atherosclerosis [17]. Under normal physiological conditions, DCs exist in the arterial wall in an immature form. In contrast, mature DCs are obviously increased in atherosclerosis plaques, mediating antigen presentation, the production of costimulation (CD80/CD86), and pro-inflammatory cytokine [IL-6, interferon-γ (IFN-γ), etc.] in the inflammatory immune microenvironment, which trigger T cell overproliferation and differentiation and exacerbate atherosclerosis eventually [18]. T lymphocytes are the major cell type regulating the adaptive immune response to atherosclerosis. They mainly include CD4^+^ T cells, regulatory T cells (T_regs_), CD8^+^ T cells, and NK T cells [19]. CD4^+^ T cells are the most plentiful T cells in mouse atherosclerotic plaques and are mainly polarized to a pro-inflammatory phenotype in the immune microenvironment, helper T cell 1 (T_H_1) cells, which secrete the inflammatory cytokines tumor necrosis factor-α (TNF-α) and IFN-γ with pro-atherosclerotic effects. In contrast, T_regs_ are thought to be atheroprotective through the secretion of IL-10 and transforming growth factor-β (TGF-β) [20,21]. Thus, immunotherapy to inhibit DC maturation, known as immune tolerance (TolDC), can inhibit the development of atherosclerosis by inducing T cell differentiation into T_regs_ and increasing anti-inflammatory cytokine (IL-10) production to reshape the immune microenvironment [22,23].

Rapamycin (RAPA), a novel macrolide immunosuppressant, can inhibit DC maturation and promote T_reg_ production [24]. However, systemic nontargeted administration of RAPA triggers many side effects, including systemic toxicity and immunosuppression, which severely limits the clinical effectiveness of immunotherapy on atherosclerosis [25]. In the face of complex physio-environment and reticuloendothelial immunological system, even nanodelivery systems labeled with targeted ligands may not be able to achieve the desired delivery efficiency [26].

**Fig. 1.**
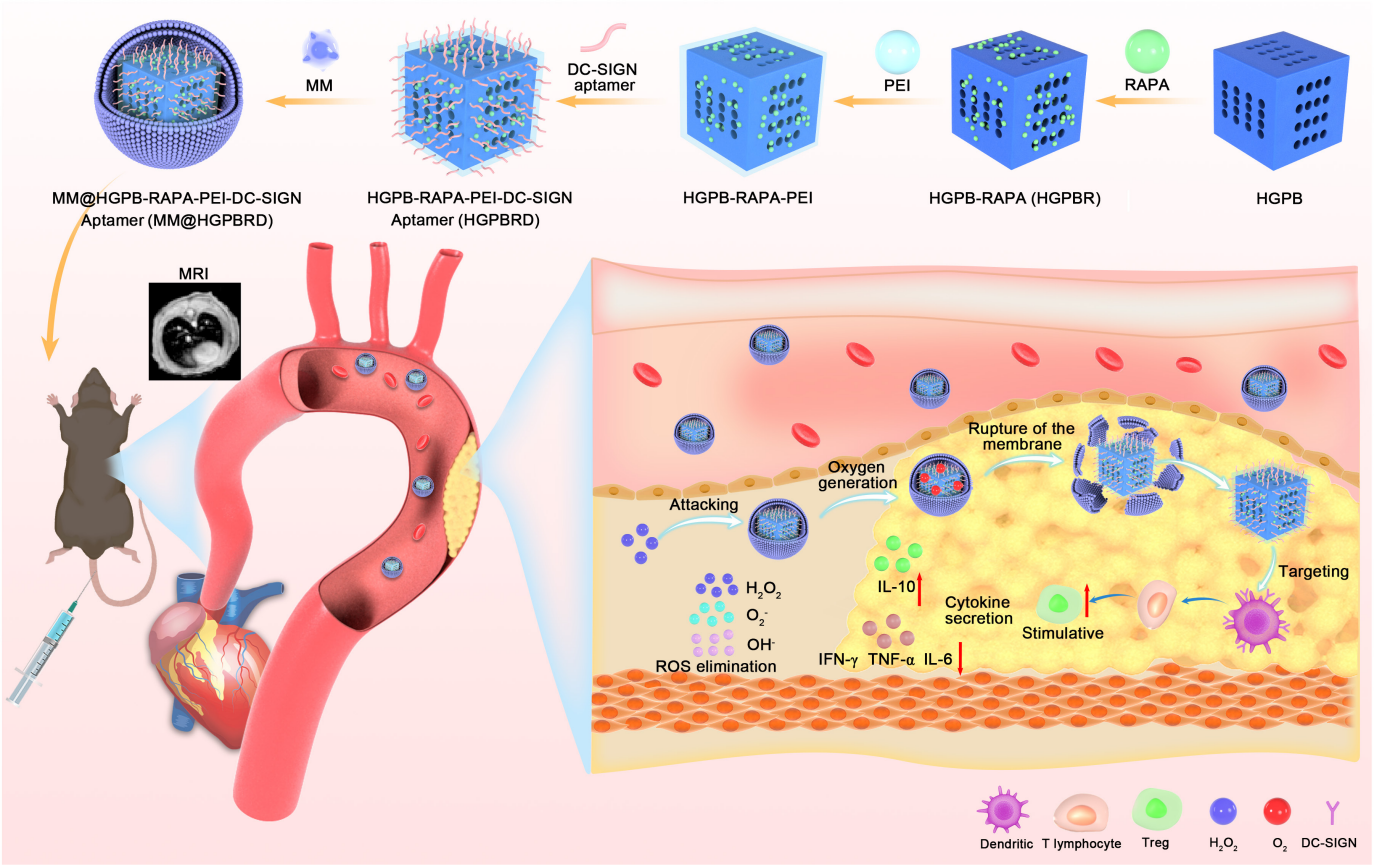
Preparation of MM@HGPBRD NPs for the inhibition and MRI of atherosclerosis.

To address this challenge, we developed a cascade targeting biomimetic nanoparticle (NP) to escape immune system clearance and target atherosclerosis lesions, and deliver RAPA to DC (Fig. 1). Gd-doped HGPB NPs was chosen as the drug nanocarrier, owing to their superior biocompatibility, structural tunability, larger inner pore volume and specific surface area, ease of synthesis and surface modification, and excellent nanoenzymatic activity [27,28]. After loading with RAPA, HGPB was further modified with DC-SIGN aptamer (HGPBRD), which can target DC cells specifically and then coated with macrophage membrane to obtain the biomimetic nanoparticle (MM@HGPBRD). The homing function of MM encapsulated on the surface of HGPBRD will enable the successful targeting and accumulation of biomimetic NPs into atherosclerotic plaques [29]. Following that, the ROS microenvironment in atherosclerotic plaques will trigger the catalase properties of HGPB to generate oxygen (O_2_), leading to membrane rupture, and exposing aptamers modified on the surface of NPs to target DCs and realize the drug delivery specifically. Atherosclerosis progression will be inhibited by the combination role of RAPA and HGPB nanozyme by decreasing DC surface costimulatory markers to inhibit DC maturation, reducing the creation of pro-inflammatory factors, contributing to the production of T_regs_ and ROS scavenging. Meanwhile, HGPB NPs with excellent magnetic resonance imaging (MRI) ability enable noninvasive real-time diagnosis of atherosclerosis plaques. Such cascade targeting strategy could effectively resolve DC targeting and realize the specific MRI of DC in atherosclerosis plaque and DC-based plaque progression inhibition.

## Materials and Methods

### Materials

Polyvinylpyrrolidone (PVP; K30), potassium ferricyanide (K_3_[Fe(CN)_6_]), PVP, and gadolinium nitrate hexahydrate [Gd(NO_3_)_3_·6H_2_O] were purchased from Dalian Meilun Biotechnology Co. Ltd. (Dalian, China). Polyethyleneimine (PEI) was obtained from Aladdin Chemical Reagent Co. Ltd. (Shanghai, China). DC-SIGN aptamer was ordered from Sangon Biotech Co. Ltd. (Shanghai, China). Polyclonal antibodies CD47 and TNFR2 were obtained from Proteintech Group Inc. (Wuhan, China). Cell Counting Kit-8 (CCK-8) cell viability/cytotoxicity assay kit was gained from Bolida Biotechnology Co. Ltd. (Xuzhou, China). Lipopolysaccharide (LPS) was purchased from Beyotime Biotechnology Co. Ltd. (Shanghai, China). Coumarin-6 (C6) was ordered from Aladdin Biochemical Technology Co. Ltd. (Shanghai, China). Fluorescent Probe-DCFH-DA was purchased from Dalian Meilun Biotechnology Co. Ltd. (Dalian, China). Phycoerythrin (PE) anti-mouse CD3, fluorescein isothiocyanate (FITC) anti-mouse CD4, PE-Cy7 anti-mouse CD25, and allophycocyanin (APC) anti-mouse FoxP3 antibodies were attained from Elabscience Biotechnology Co. Ltd. (Wuhan, China). Mouse IL-10, IFN-γ, IL-6, and TNF-α enzyme-linked immunosorbent assay (ELISA) kits were received from Jianglai Bio Co. Ltd. (Shanghai, China).

### Synthesis of HGPB

HGPB NPs were fabricated by a hydrothermal method as our previous report in literature [30,31]. First, 4 g of PVP and 128 mg of K_3_[Fe(CN)_6_] were diffused in 40 ml of 0.01 M HCl and the mixture was swirled for 30 min to form liquid A. Four grams of PVP and 240 mg of Gd(NO_3_)^.^6H_2_O were diluted in 40 ml of 0.01 M HCl and agitated for 30 min to form liquid B. Then, liquid A and B were whisked together for 3 h to obtain a clarified yellow solution and reacted at 80 °C for 24 h. Afterward, reaction solution was centrifuged at 16,000 rpm for 30 min, and precipitates were washed several times with ultrapure water or ethanol, respectively, driven to dryness under ambient temperature to obtain GPB crystals with cubic morphology. To prepare HGPB NPs, 175 mg of PVP and 35 mg of GPB were solubilized in 35 ml of 1 M HCl and swirled for 3 h and reacted for 3 h at 140 °C. After centrifugation at 16,000 rpm for 30 min, the obtained sediment was cleaned twice with ultrapure aqueous and once with ethanol and dried at room temperature to obtain HGPB.

### Drug loading

In order to prepare RAPA-loaded HGPB (HGPBR), HGPB and RAPA were dispersed in a dimethyl sulfoxide (DMSO) solution with a mass proportion of 10:1, 5:1, 2:1, 1:1, 1:2, 1:5, 1:8, 1:10, and 1:12, agitated at ambient temperature under light protection for 24 h. Precipitation was acquired by centrifugation at 16,000 rpm for 30 min and washed with ultrapure aqueous for several times to eliminate unloaded RAPA.

A standard fitting curve relating concentration to absorbance of RAPA was established by ultraviolet–visible (UV–vis) spectrophotometer. The maximum UV–vis absorption peak of RAPA was 280 nm. The mass of RAPA in the upper layer of liquid was performed with the standard fitting curve. The drug loading capacity (LC) and encapsulation efficiency (EE) were estimated by the following formulas:

$$\begin{array}{c} \mathrm{LC}\;\left(\mathrm{wt}\%\right)=\frac{\;\text{mass of drug loaded in the final carriers}}{\text{mass of the drug loaded final carriers}}\times 100\% \end{array}$$ (1)

$$\begin{array}{c} \mathrm{EE}\;\left(\mathrm{wt}\%\right)=\frac{\;\text{mass of drug loaded in the final carriers}}{\text{mass of drug}\;fed\;\text{initially}}\times 100\% \end{array}$$ (2)

### Preparation of HGPBRD

For the preparation of HGPBRD, PEI was first modified on the surface of HGPBR. In brief, 10 ml of PEI (0.2 mg/ml) was dropped to 10 ml of HGPBR dispersion (0.5 mg/ml) and agitated for 1 h. Liquid mixture was then decanted by centrifugation (16,000 rpm, 30 min), and the sediment was taken out and purified several times to capture the PEI-modified HGPBR. Then, DC-SIGN aptamer solution (10 μl, 100 μM) was added drop by drop. After further magnetic stirring in an ice-water bath for 4 h, the reaction agent was removed and centrifuged at 12,000 rpm for 20 min to achieve precipitate, which was washed with ultrapure aqueous to successfully get HGPBRD. The NP concentrations were quantitatively determined through a standardized drying-weighing protocol following synthesis.

### Isolation of MM

Briefly, 8 × 10^8^ macrophages were collected and washed with phosphate-buffered saline (PBS) (500 g for 10 min each time), and the cells were precipitated in hypotonic buffer lysate containing 1 mM NaHCO_3_, 0.2 mM ethylene diamine tetraacetic acid (EDTA), and 1 mM phenylmethylsulfonyl fluoride (PMSF). Then, cell lysate was resuspended well before incubation at 4 °C overnight. Subsequently, the cell lysate was loaded into an automated ultrasonic crusher and disrupted for 60 cycles. The cell solution was then centrifuged at 3,200*g* for 5 min at 4 °C to eliminate particles, and the upper layer of liquid was gathered. After another 25 min of centrifugation at 20,000*g*, 4 °C, the sediment was abandoned and the upper layer of liquid was gathered. The supernatant was abandoned after centrifugation at 100,000*g* for another 35 min at 4 °C, the precipitate (MMs) was gathered and resolubilized in PBS, and the macrophage protein content in the membrane was quantified by bicinchoninic acid (BCA) protein assay.

Next, MM suspension was extruded back and forth several times through the liposome extruder (Avanti, USA) inside the liposome extruder to get MM vesicles. The resultant MM vesicles were placed at 4 °C.

### Preparation of MM@HGPBRD

Coating of MMs on the surface of HGPBRD was achieved by extrusion. In short, HGPBRD NPs were combined with purified MMs (weight ratio between NPs to membrane proteins = 1:1) and then extruded through a 400-nm polycarbonate membrane to achieve MM@HGPBRD. The prepared MM@HGPBRD NP solvent was saved at 4 °C for later use.

### Characterization of the NPs

The morphology of the above formulated NPs was observed by transmission electron microscopy (TEM; JEM-1230, Japan). The hydrodynamic sizes and potentials were detected by Zetasizer Nano ZS90 (Malvern Instruments, UK). The pore size distribution and specific surface area of the NPs were determined by Brunauer–Emmett–Teller (BET) method (ASAP 2460 3.01, Beijing, China). To verify the successful synthesis of MM@HGPBRD, RAPA, HGPB, HGPBR, HGPBRD, MM vesicles, and MM@HGPBRD, UV–vis spectroscopy (UH4150, Japan) and Fourier transform infrared (FTIR) spectroscopy (TENSOR27, Germany) were employed to characterize the preparation of MM@HGPBRD. The protein composition of the cell membrane of MM@HGPBRD was demonstrated by sodium dodecyl sulfate–polyacrylamide gel electrophoresis (SDS-PAGE) and Coomassie blue staining. The attachment of DC-SIGN aptamer in MM@HGPBRD was verified by agarose gel electrophoresis.

### Confirmation of oxygen generation and cell membrane rupture in vitro

To verify the ability of MM@HGPBRD to produce oxygen, HGPB, HGPBR, HGPBRD, and MM@HGPBRD NPs were first prepared. Two milliliters of 0.02 mg/ml of them was mixed with 200 μl of H_2_O_2_ (100 μM) and reacted for 10 min. Oxygen production in individual sample was detected by portable dissolved oxygen meter (JPSJ-605F, Shanghai, China). The effect of PBS on oxygen production was determined under the same conditions.

To verify cell membrane rupture, MM@HGPBRD was dispersed in PBS (pH 6.0, containing 100 μM H_2_O_2_) or PBS (pH 7.4) for 2-min sonication and treated for 1 h. The solution was taken and added dropwise to a copper grid and then dried naturally. The membrane rupture of MM@HGPBRD was observed by TEM.

### Drug release behavior

To study RAPA release, MM@HGPBRD and HGPBRD were dispersed in 1 ml of PBS buffer containing 0.2% Tween 20 with different pH values (pH 6.0 with or without 100 μM H_2_O_2_, or pH 7.4) and shaken at 100 rpm at 37 °C. Then, at 1, 2, 4, 6, 8, 12, 24, and 48 h, the solution was centrifuged and 1 ml of upper layer of liquid was taken out and 1 ml of PBS containing 0.2% Tween 20 was replenished. The released drug in supernatant was assayed by UV–vis spectrophotometer, and the cumulative percentage of drug release was counted.

### Stability test

MM@HGPBRD was dissolved in PBS (pH 7.4) and stored for 1 week. Samples were collected at predetermined time points (0, 1, 3, 5, and 7 d) to evaluate storage stability. Particle size variations were measured using a Zetasizer Nano ZS90 analyzer (Malvern Instruments, UK). Meanwhile, the corresponding samples were also performed with SDS-PAGE as the above method to assess the stability of DC-SIGN aptamer in MM@HGPBRD samples.

### Characterization of nanozyme activity

To measure the catalase property-like activity, MM@HGPBRD NPs or HGPB NPs with different concentrations (0, 20, 50, 80, 100, and 200 μg/ml) were dispersed in 2 ml of PBS (pH 7.4) containing H_2_O_2_ (500 mM) and reacted for 2 h. Subsequently, the absorbance at 240 nm was evaluated by a NanoDrop One/OneC Microvolume UV-vis Spectrophotometer (Thermo Fisher Scientific) to assess their ability to clear H_2_O_2_.

Their peroxidase-like activity was investigated employing the 3,3′,5,5′-tetramethylbenzidine (TMB) chromogenic method [31]. PBS buffer (200 μl, pH 6.0), MM@HGPBRD NPs or HGPB NPs (5 μl, 20 μg/ml), TMB (10 μl, 10 mg/ml), and 20 μl of 30% H_2_O_2_ solution were placed to a 96-well plate, and then the difference in absorbance of the solution at 650 nm over a period of 5 min was tested by a microplate reader (SpectraMax i3, Molecular Devices, USA). Similarly, different concentrations of MM@HGPBRD NPs or HGPB NPs were mixed and reacted with the above solutions for 5 min, and the absorbance of the liquid at 650 nm was recorded (*n* = 3).

To determine the superoxide dismutase-like activity, MM@HGPBRD NPs or HGPB NPs (50 μg/ml) were placed to PBS buffer solution (pH 7.4) containing adenine (1 mM). Then, 50 μl of mixture was transferred to the measurement chamber. Subsequently, electron paramagnetic resonance was used to detect superoxide anion (O_2_^−^) in different reaction solutions.

### Determination of MRI relaxivity of MM@HGPBRD

The T_1_-weighted MRI relaxivity of MM@HGPBRD NPs was performed at 3.0T MR system (GE 750 W, USA). T_1_ images of MM@HGPBRD NPs with different concentrations (Gd^3+^ concentrations ranging from 0 to 0.020 mM) were obtained by 3.0T MRI, and the T_1_ relaxation times of the corresponding images were tested. The horizontal coordinate was the concentration of Gd, and the vertical coordinate was the reciprocal of the T_1_ relaxation time of the sample. T_1_ relaxation rate was expressed as the slope of the linear regression equation. The scanning parameters were set as follows: field of view = 18 cm × 18 cm, echo time = min full, repetition time = 425 ms, matrix size = 384 × 224, slice spacing = 1.5 mm, slice thickness = 3.0 mm.

### Cells and cell culture

Mouse dendritic cells (DC2.4), mouse macrophages (RAW264.7), and mouse embryonic fibroblasts (3T3) were purchased from the Cell Bank of the Chinese Academy of Sciences. All of the above cells were cultured in Dulbecco’s modified Eagle’s medium (DMEM) containing fetal bovine serum (10%), penicillin (100 U/ml), and streptomycin (0.1 mg/ml) in an incubator at 37 °C with 5% CO_2_.

### Cytotoxicity assessment

The CCK-8 method was adopted to assess the cytotoxicity of HGPBRD NPs or MM@HGPBRD NPs in vitro. Briefly, DC2.4, RAW264.7, and 3T3 cells (1 × 10^4^ cells per well) were inoculated in 96-well plates and incubated at 5% CO_2_, 37 °C for 24 h (*n* = 3). Subsequently, the culture medium was taken out and 100 μl of medium with various concentrations of HGPBRD NPs or MM@HGPBRD NPs (0, 50, 80, 100, 150, 200, 250, 300, and 400 μg/ml) was added and cultured for another 24 h. After eliminating the medium and cleaning cells with PBS buffer, DMEM containing CCK-8 (10%) was placed to each cell well and incubated for 1 to 4 h away from light. Absorbance at 450 nm was taken by a microplate reader (SpectraMax i3, Molecular Devices, USA).

### In vitro cellular uptake and endo/lysosome escape study

For fluorescence imaging by confocal laser scanning microscope (CLSM; Leica STELLARIS 5, Germany), 1 wt % C6 was first primed to HGPB, HGPBR, HGPBRD, and MM@HGPBRD. Subsequently, DC2.4 cells were inoculated in 6-well plates (1 × 10^4^/well) and cultured overnight, and cell maturation was induced with LPS (100 ng/ml). Then, HGPB, HGPBR, HGPBRD, and MM@HGPBRD with C6 loading were placed and individually incubated for 2 h. The solutions were abandoned, and cells were washed with PBS and immobilized with 4% paraformaldehyde. Cell nuclei were stained with 4′,6-diamidino-2-phenylindole (DAPI) staining solution containing anti-fluorescent quencher. Lastly, cells were viewed and photographed with confocal laser scanning microscope (CLSM).

To verify the role of MM encapsulation in evading immunological clearance, RAW264.7 cells were seeded in 6-well plates at a concentration of 1 × 10^4^ cells in each well and grown overnight. After incubation with C6-loaded HGPBRD or MM@HGPBRD for 0.15, 0.5, 1, and 2 h, the cells were washed with PBS and immobilized with paraformaldehyde. Afterward, the nuclei were dyed with DAPI. The phagocytosis of NPs by macrophages was visualized through the CLSM.

For quantitative analysis, flow cytometry characterization was performed. Briefly, DC2.4 cells (1 × 10^5^ cells/well) were seeded in 6-well plates and grown overnight with LPS (100 ng/ml) to activate DCs. C6-loaded HGPB, HGPBR, HGPBRD MM@HGPBR, or MM@HGPBRD were added and incubated for 2 h. To further study the time influence, C6-loaded MM@HGPBRD was incubated with activated DC2.4 cells for 0, 10, 20, 30, 60, and 120 min. The treated cells were finally tested by flow cytometry (FACSCanto II, USA).

### Assessment of DC2.4 activation and maturation in vitro

DC2.4 cells (2 × 10^5^ cells) were inoculated in 6-well plates and incorporated at 37 °C and 5% CO_2_ for 24 h. RAPA (10 nM), HGPB, HGPBR, HGPBRD, and MM@HGPBRD (20 μg/ml) were supplemented after incubation with the cells for 2 h. Cell maturation was then induced by adding LPS (100 ng/ml) to the remaining cell wells except the blank wells. Ultimately, the cells were gathered and colored with the antibody mixtures (PE anti-CD11c, FITC anti-CD80, and APC anti-CD86) at 4 °C for 35 min and were detected by flow cytometry (*n* = 3).

### In vitro cytokine secretion

To confirm the in vitro anti-inflammatory effect, DC2.4 cells in a 6-well plate were matured by the addition of LPS (100 ng/ml) except the blank wells. Then, HGPB, HGPBR, HGPBRD, and MM@HGPBRD (20 μg/ml) were placed to incubate with the cells for 2 h. Subsequently, the cell supernatant of each cell well was aspirated and the development of pro-inflammatory cytokines (TNF-α, IFN-γ, and IL-6) and anti-inflammatory cytokines (IL-10) expression in the cell supernatant was tested by ELISA.

### ROS scavenging at the cellular level

DC2.4 cells were grown overnight in 6-well plates at a concentration of 1 × 10^5^ per well. Cells in the control group were cultivated in medium, and cells in the model group were treated with LPS (100 ng/ml) for 1 h. Afterward, different groups of cells were cocultured with HGPB, HGPBR, HGPBRD, and MM@HGPBRD NPs at a consistency of 20 μg/ml for 2 h, separately. Afterward, the cells were flushed and processed with DCFH-DA (10 μM) in PBS for 30 min. Following cleaning with PBS, ROS creation in DC2.4 cells was tested by inverted fluorescence microscopy. For quantitative test of ROS production, the above groups of cells were ablated down and resuspended in 4% paraformaldehyde and performed by flow cytometry.

### Targeted MRI in vitro

DC2.4 cells were grown in 6-well plates (1 × 10^5^ cells per well) and incorporated for 12 h with or without LPS (100 ng/ml) induction. Then, different groups of cells were incubated with HGPB, HGPBR, HGPBRD, and MM@HGPBRD NPs for 2 h. Afterward, the cells were washed with PBS, digested with trypsin, and centrifuged. The collected cells were solubilized in 4% paraformaldehyde solution and centrifuged again to make the cells uniformly concentrated at the bottom of the centrifuge tubes for MRI on a 3.0T MR scanner (GE 750 W, USA). PBS-treated DCs served as a blank control group. Finally, MRI of the cells was performed and the T_1_ signal strength of each group was recorded.

### Animals and model establishment

Animal experiments were conducted in accordance with the National Institutes of Health (NIH) guidelines for the use of animals in research. Female C57BL/6 mice were purchased from the Animal Center of Xuzhou Medical University. Eight-week-old female apolipoprotein E knockout (ApoE^−/−^) mice were gained from Cavens Experimental Animal Co. Ltd. (Changzhou, China). All animals were kept with standard rearing culture, and all animals were acclimatized to the environment for a minimum of 3 d before the initiation of the trials. All animal test protocols adhered to the NIH Guide for the Care and Use of Laboratory Animals and ethically endorsed by the Ethical Committee of Xuzhou Medical University (202306T025). ApoE^−/−^ mice were given a high-fat diet containing 40% fat, 1.25% cholesterol, and 0.5% sodium cholate for 2 months to build an animal model of atherosclerosis.

### In vivo safety evaluation

To study the in vivo toxicity of MM@HGPBRD NPs, 12 C57BL/6 mice (female, 6 weeks) were randomly divided into 4 groups. Saline or MM@HGPBRD NPs (5 mg/kg) were then injected by caudal vein. The collection of blood samples for routine blood analysis and blood biochemistry analysis was performed on days 1, 7, and 21 after injection. At the termination of blood collection, mice were dissected to collect organs such as heart, liver, spleen, kidney, and lungs, which were fixed in 10% formalin, sectioned, and stained with hematoxylin and eosin (H&E) for histological analysis. Eventually, observation was performed using a light microscope.

To evaluate the blood compatibility of MM@HGPBRD NPs, after washing 1 ml of mouse blood with physiological saline 3 times, 200 μl of erythrocytes was diluted with 10 ml of isotonic sodium chloride solution. The diluted blood sample (0.2 ml) was added to 800 μl of MM@HGPBRD NP solution at different concentrations (10, 50, 80, 100, 150, 200, 250, 300, and 400 μg/ml), and saline-treated and deionized water-treated erythrocytes were used as a negative and positive control, respectively. The solutions were incubated at 37 °C for 2 h and centrifuged at 3,000 rpm for 5 min. The upper layer of liquid from each group was taken out and placed to a 96-well plate to measure the absorbance at 540 nm by a microplate reader to determine the hemoglobin released from the lysed erythrocytes. The sample hemolysis rate was used as the following formula:

$$\begin{array}{c} \text{Hemolysis}\;\left(\%\right)=\frac{\text{ODsample}\;-\;\text{ODnegative contro}\text{l}}{\text{ODpositive}\;-\;\text{ODnegative contro}\text{l}}\times 100\% \end{array}$$ (3)

In vivo targeted imaging of atherosclerosis: When ApoE^−/−^ mice were successfully modeled, mice were administered with HGPBRD NPs or MM@HGPBRD NPs (5 mg/kg) via caudal vein injection for in vivo MRI. Whole-body images of mice were obtained at 0, 1, 2, 4, 6, 8, 10, 12, and 24 h before and after NP administration on a 3.0T MRI system (GE 750W, USA). The regions of interest (ROIs) were the aortic region and metabolic organs. The T_1_ signal strength at each time point was taken on an AW4.6 postprocessing workstation.

### In vivo anti-atherosclerosis therapy

ApoE^−/−^ mice were divided into 7 groups: (I) control group, (II) model group, (III) RAPA group, (IV) HGPB group, (V) HGPBR group, (VI) HGPBRD group, and (VII) MM@HGPBRD group (*n* = 6). Control mice were treated with a normal diet, and mice in the other groups were given a high-fat diet for 2 months. Afterward, mice in groups III to VII were injected weekly with 200 μl of RAPA (2.4 mg/kg), HGPB NPs, HGPBR NPs, HGPBRD NPs, and MM@HGPBRD NPs (5 mg/kg) intravenously by tail vein injection. Mice in group II were injected intravenously with 200 μl of saline. Mice were subjected to weekly treatments and kept on a high-fat diet during this period, and their body weights were recorded weekly. After 8 weeks, all mice were subjected to euthanasia and aortas were gathered for oil red O staining and quantified by ImageJ to assess the treatment efficacy of the different groups.

### Histology and immunohistochemistry

For histological analysis, aortas from the above 7 groups were gathered for H&E staining and toluidine blue staining. The vessel area and plaque area were measured manually using ImageJ software. For immunohistochemical analysis, aortic sections from each of the 7 groups were incubated with antibodies, including CD11c, CD80, CD86, and Foxp3, and then positively stained cells were quantified by ImageJ software.

### Assessment of inhibition of DC maturation and stimulation of T_reg_ responses

Shortly, spleens and lymph nodes of euthanized mice from each group (*n* = 3) after different treatments were removed and grounded on ice into cell suspensions. Subsequently, the cell suspensions were incubated with antibodies (PE anti-CD11c, FITC anti-CD80, APC anti-CD86, PE anti-CD3, FITC anti-CD4, PE Cy7 anti-CD25, and APC anti-Foxp3) at 4 °C for half an hour and then detected by flow cytometry.

### In vivo cytokine analysis

To verify the in vivo anti-inflammatory effect, orbital blood was gathered from the eyes of mice in 7 groups at termination of therapy, and the serum was subsequently centrifuged and the expression of TNF-α, IFN-γ, IL-6, and IL-10 in the serum of mice was determined by ELISA.

### Statistical analysis

All figures were expressed as mean ± SD and statistically analyzed with GraphPad Prism 8.0 software and Student’s *t* test, one-way analysis of variance (ANOVA) method. Differences were significant when *P < 0.05 and highly significant when **P < 0.01 or ***P < 0.001.

## Results and Discussion

### Preparation and characterization of MM@HGPBRD

**Fig. 2.**
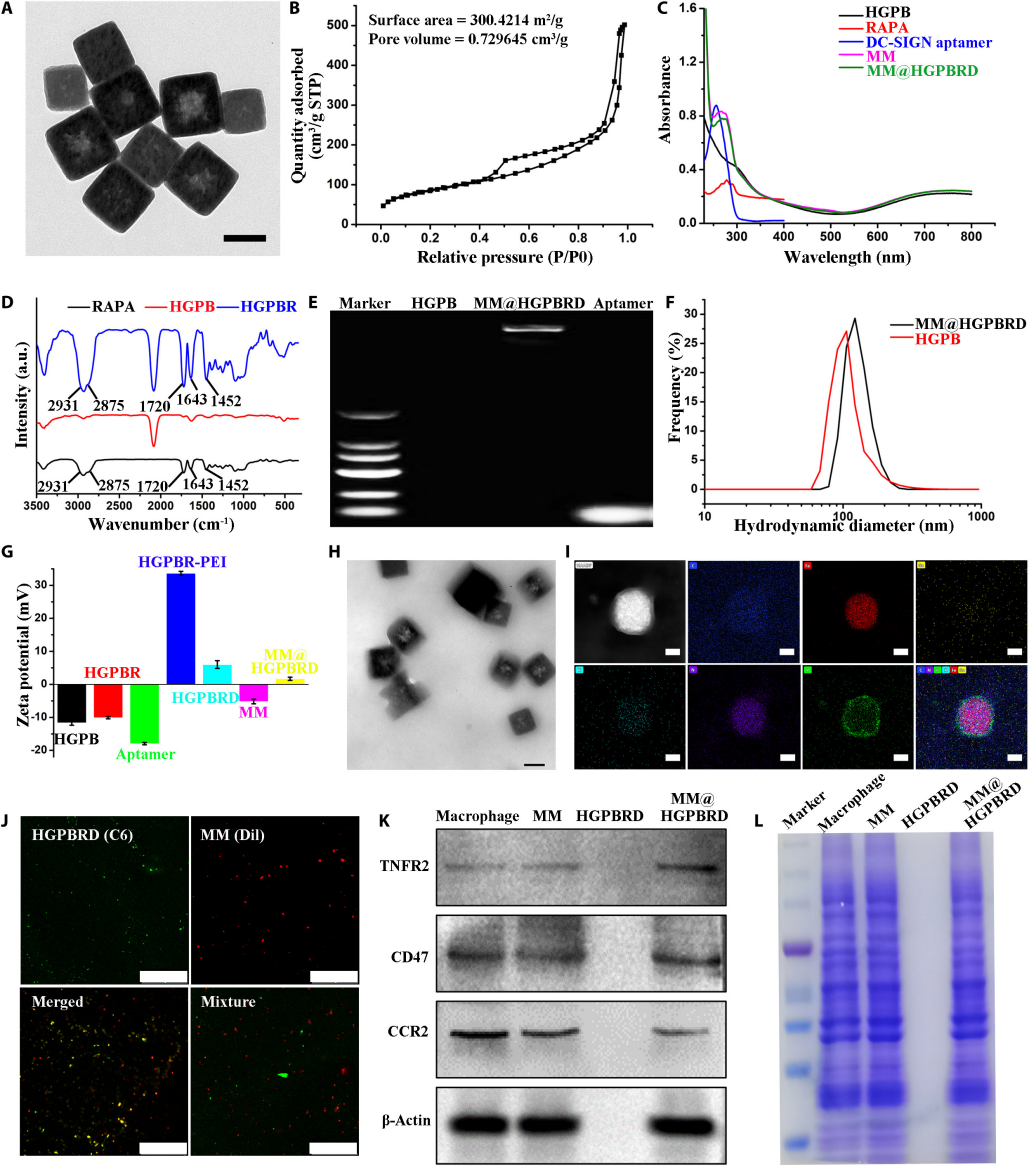
Characterization of MM@HGPBRD. (A) TEM images of HGPB (scale bar, 100 nm). (B) N_2_ adsorption/desorption isotherms of HGPB. (C) UV–vis spectra of HGPB, RAPA, DC-SIGN aptamer, MM, and MM@HGPBRD. (D) FTIR spectra of RAPA, HGPB, and HGPBR. (E) Gel retardation assay for DC-SIGN aptamer modification. (F) Hydrodynamic size and (G) zeta potentials of different NPs. (H) TEM images of MM@HGPBRD (scale bar, 100 nm). (I) Element mapping of MM@HGPBRD (scale bar, 50 nm). (J) Confocal images of C6-loaded HGPBRD (green) and Dil-stained MM (red) (scale bar, 50 μm). (K) Western blot assay of specific protein markers of macrophage, MM, HGPBRD, and MM@HGPBRD. (L) SDS-PAGE gel electrophoresis analysis of MM@HGPBRD.

To fabricate the spontaneous induced cascade targeting biomimetic NPs for the diagnosis and therapy of atherosclerosis, gadolinium-doped Prussian blue (GPB) was first synthesized by hydrothermal method and etched in hydrochloric acid to form a hollow mesoporous structure (HGPB) [30]. The hollow mesoporous structures of HGPB were characterized by TEM. TEM images showed that the constructed HGPB was monodispersed, cubic shaped, and homogeneous in size at ~113 nm (Fig. 2A). N_2_ absorption isotherms of HGPB were obtained by the BET method. It was shown that HGPB had a surface area of about 300.4 m^2^/g and an average pore size of about 10.1 nm (Fig. 2B), which provided space to efficiently encapsulate small drug molecules into the pores. Subsequently, the drug RAPA was loaded in HGPB to obtain HGPBR. The RAPA encapsulation and LCs were optimized with different mass ratios between HGPB and RAPA. As shown in Table S1, with the increase of RAPA amount, higher drug LC was obtained and the corresponding encapsulation rate was increased to a relative stable state and then decreased. Considering the balance between nanocarrier amount and drug effective concentration [32], a 1:2 mass ratio was selected in the following study with loading and encapsulation rates of 46.2% and 48%, respectively. The UV–vis absorption spectra exhibited featured absorption peak of RAPA at ~280 nm (Fig. 2C). Similarly in the FTIR spectra, there were featured absorption peaks of RAPA at 1,452, 1,643, 1,720, 2,875, 2,931, and 2,965 cm^−1^ (Fig. 2D), indicating the successful loading of RAPA in HGPBR. To further endow HGPBR with targeting ability to DC cells, we modified the surface of HGPBR with PEI. Subsequently, negatively charged DC-SIGN aptamers were loaded onto the surface of positively charged PEI-HGPBR NPs by electrostatic adsorption. The UV–vis spectra exhibited the featured absorption peak of the DC-SIGN aptamer at ~260 nm (Fig. 2C). To avoid clearance of the endothelial reticular system and actively home to atherosclerosis lesions, MMs were extracted and coated with HGPBRD NP by extrusion through a liposome extruder to obtain MM@HGPBRD NPs. Agarose gel electrophoresis results displayed no obvious characteristic bands for HGPB, whereas bright bands appeared for DC-SIGN aptamer and MM@HGPBRD. Furthermore, MM@HGPBRD had an increased molecular mass compared to the aptamer alone so that the bright bands were still retained in the sample wells (Fig. 2E). The dynamic light scattering (DLS) (Fig. 2F) and zeta potential (Fig. 2G) results also testified such successful fabrication process. It was exhibited that the hydrodynamic diameter of HGPB NPs was 101.8 ± 0.6 nm and the zeta potential was −11.6 ± 0.7 mV. After drug loading, PEI modification, DC-SIGN aptamer attachment, and MM coating, the hydrodynamic size of HGPB NPs increased to 125.5 ± 0.7 nm for MM@HGPBRD and the zeta potential was changed from 10.1 ± 0.3 mV for HGPBR to 33.6 ± 0.6 mV for HGPBR-PEI, 6 ± 1.2 mV for HGPBRD, and finally 1.7 ± 0.5 mV for MM@HGPBRD. After MM coating, the obvious MM shell layer on the surface of MM@HGPBRD could be identified from TEM image (Fig. 2H). Elemental mapping analysis displayed the elemental composition and distribution of MM@HGPBRD, and the results revealed not only the elements C, N, K, Fe, and Gd but also the MM characteristic element, P (Fig. 2I), further confirming the successful coating of MM. Furthermore, we labeled MM with Dil (red) and HGPBRD with C6 (green). As shown in Fig. 2J, confocal microscopy imaging results demonstrated the colocalization of HGPBRD (green) and MM (red). Additionally, key membrane antigens such as TNF receptor 2 (TNFR2), CD47, and CCR2 [receptor for monocyte chemoattractant protein-1 (MCP-1)] were preserved on the surfaces of both MM and MM@HGPBRD testified by Western blot analysis (Fig. 2K), suggesting the relative integrity of MM on MM@HGPBRD. Coomassie blue staining presented similar result (Fig. 2L), providing the basis for the immune system clearance escape and active homing ability of MM@HGPBRD, which was ascribed to the function of MM. Furthermore, during the storage at 4 °C for 7 d, their hydrodynamic size and the band on the gel changed only a little, showing the relative stability of MM@HGPBRD (Fig. S1).

### Nanozyme properties and drug release promotion of biomimetic NPs

**Fig. 3.**
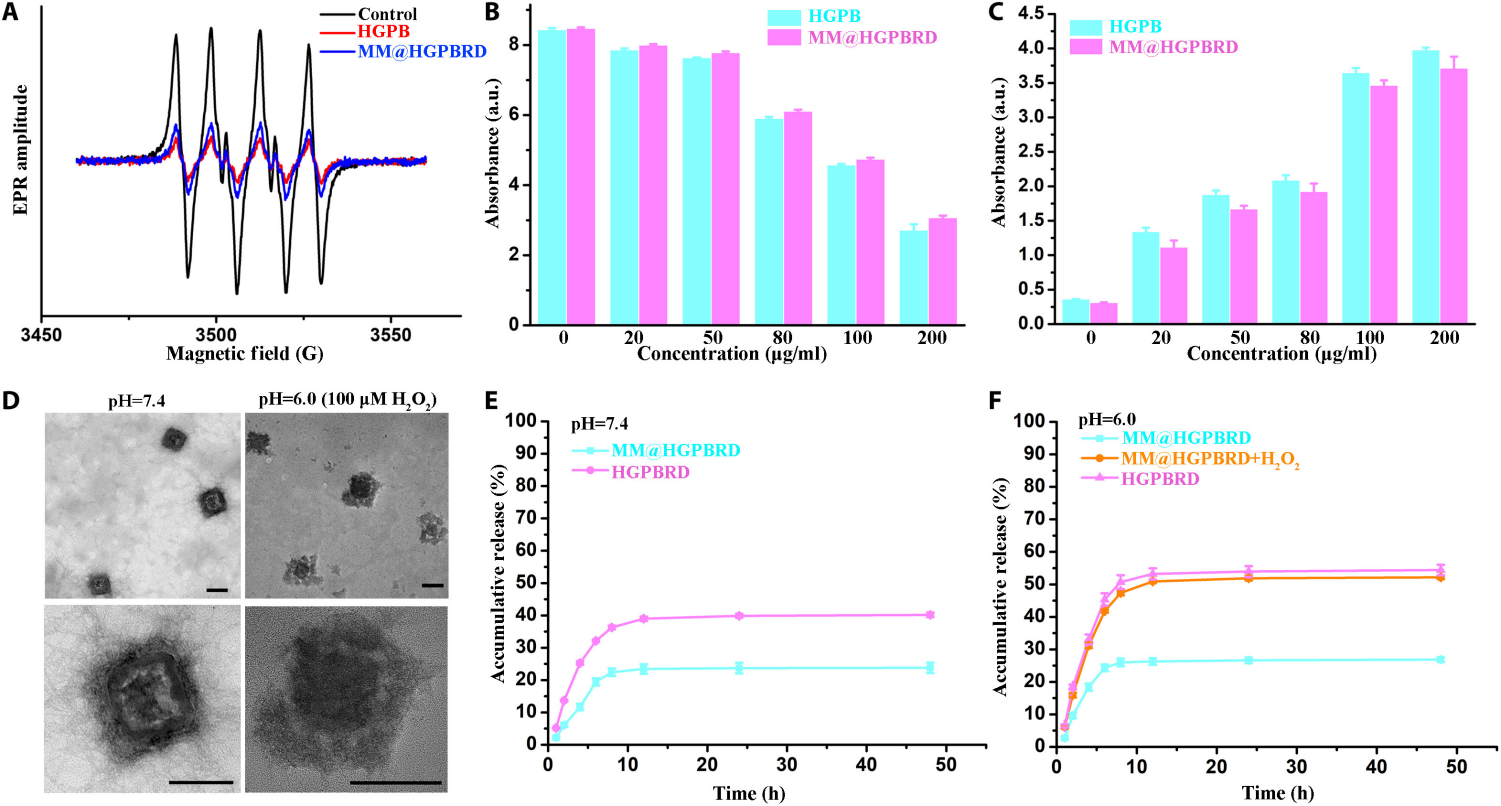
Characterization of MM@HGPBRD properties. (A) Electron paramagnetic resonance measurement of superoxide dismutase activities of HGPB and MM@HGPBRD. Measurement of catalase property-like (B) and peroxidase-like (C) activities of HGPB and MM@HGPBRD at different concentrations. (D) TEM of cell membrane rupture of MM@HGPBRD under different conditions (scale bar, 100 nm). (E and F) Drug release of HGPBRD and MM@HGPBRD under different environments.

PB NPs were reported to possess multi-enzyme activities such as peroxidase, catalase properties, and superoxide dismutase properties, which could effectively scavenge ROS for their applications in many diseases such as tumor, ischemic stroke, and atherosclerosis [27,28]. Thus, in this work, we would like to combine the nanozyme activity of HGPB with RAPA to inhibit the progression of atherosclerosis. To study the influence of drug loading, aptamer modification, and MM coating on nanozyme activity, we assessed and compared the superoxide dismutase, catalase properties, and peroxidase properties of HGPB and MM@HGPBRD. First, O_2_^−^ is one of the ROS that can be eradicated by superoxide dismutase. Since the xanthine/xanthine oxidase system is capable of inducing the production of O_2_^−^, we chose the xanthine/xanthine oxidase system to study the superoxide dismutase-like activity of HGPBRD and MM@HGPBRD. From the results of Fig. 3A, the electron paramagnetic resonance amplitudes of 5,5-dimethyl-1-pyrroline N-oxide (DMPO)/O_2_^−^ of HGPBRD and MM@HGPBRD were reduced to different degrees in comparison to the control group, suggesting a higher O_2_^−^ scavenging ability of HGPBRD and MM@HGPBRD.

Subsequently, catalase properties and peroxidase enzyme activities were assayed for HGPB NPs and MM@HGPBRD NPs according to previous literature [31]. It was reported that 1 mM H_2_O_2_ was a typical ROS concentration close to the oxidative stress in atherosclerotic plaques [33,34]. Also, 0.1 to 1 mM have been commonly used as the simulated level of H_2_O_2_ in vitro for atherosclerotic lesion [29,35]. Thus, 100 μM H_2_O_2_ was used for the evaluation of H_2_O_2_-scavenging nanozyme activity of MM@HGPBRD. As shown in Fig. 3B and C, HGPB NPs and MM@HGPBRD NPs showed excellent catalase properties and peroxidase enzyme activities and induced H_2_O_2_ depletion in a dose-dependent manner. It should be mentioned that the MM coating did not have an obvious interference on the catalase properties, peroxidase, and superoxide dismutase-mimicking catalytic activities of the HGPB nanozymes. In addition, all of the NPs (HGPB, HGPBR, HGPBRD, and MM@HGPBRD) could catalyze the decomposition of H_2_O_2_, leading to the production of O_2_, and its amount was increased with the time passing by (Fig. S2). MM@HGPBRD and HGPB were able to generate 12.15 and 12.17 mg/l of oxygen in 1 min in water, respectively, which provided an opportunity for the cascade targeting.

In previous studies, gases were used to rupture membranes to achieve drug release [36,37]. Thus, we evaluated the feasibility of membrane disruption of MM@HGPBRD by the generated oxygen to realize cascade targeting design. As shown in Fig. 3D, compared with the smooth surface of MM@HGPBRD in pH 7.4 PBS buffer, MM@HGPBRD in pH 6.0 PBS buffer containing 100 μM H_2_O_2_ displayed an irregular surface from TEM images, showing the damaged and ruptured membrane of MM@HGPBRD with the production of oxygen. Such membrane rupture could not only expose DC-SIGN aptamer but also accelerate the drug release. As shown in Fig. 3E, MM coating could protect RAPA leakage during their circulation in blood. But in mimic atherosclerosis plaque microenvironment (pH 6.0, H_2_O_2_), the presence of H_2_O_2_ destroyed the cell membrane of MM@HGPBRD and promoted RAPA release in the lesion region (Fig. 3F).

### ROS-scavenging and anti-inflammatory effect in activated DC2.4 cells

**Fig. 4.**
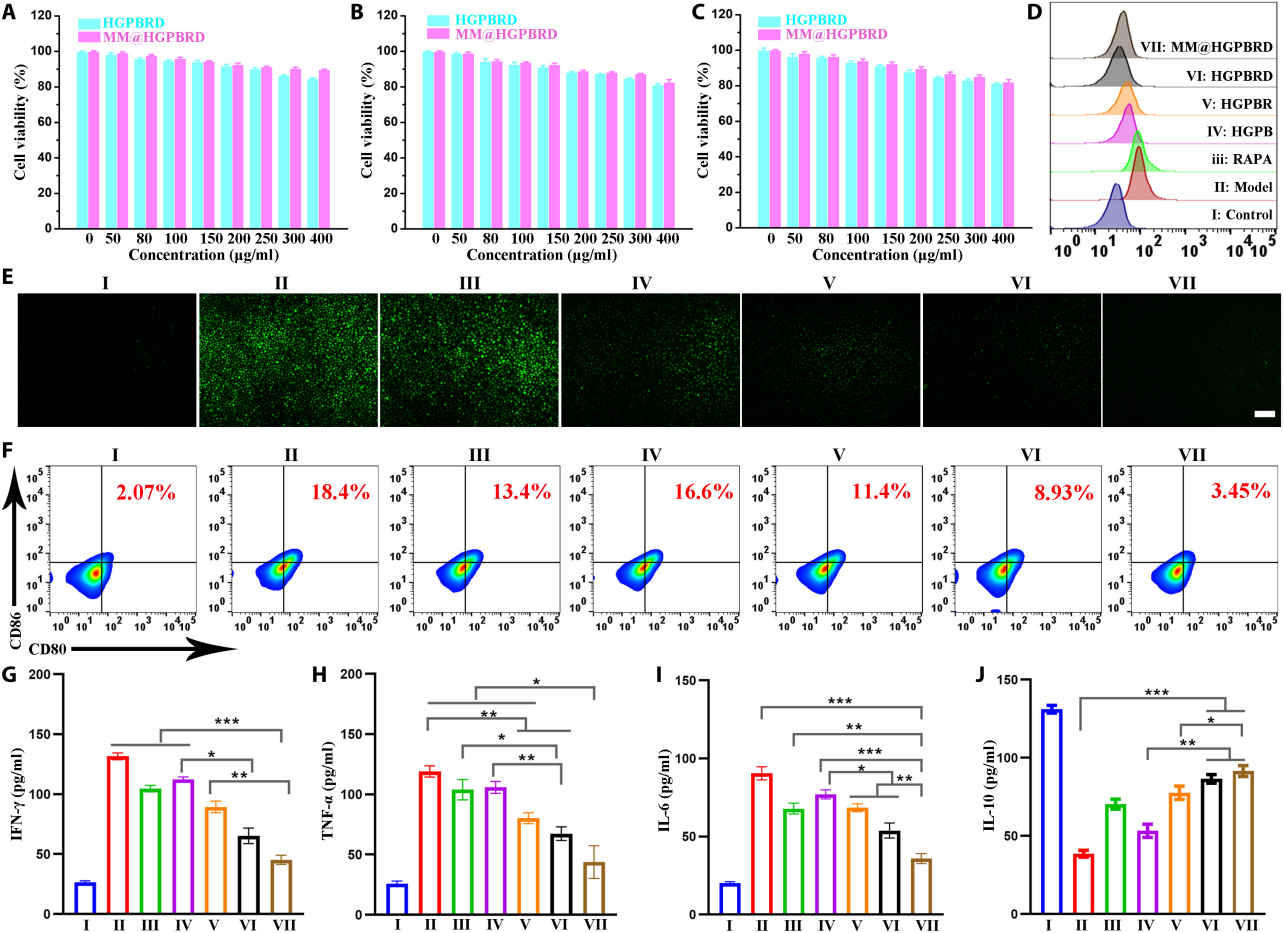
In vitro functional verification of MM@HGPBRD. Toxicity of HGPBRD and MM@HGPBRD with different concentrations on DC2.4 (A), RAW264.7 (B), and 3T3 (C). (D) Flow cytometry detection of ROS production in DC2.4 after different treatments. (E) Fluorescence images of ROS production in DC2.4 after different treatments (scale bar, 200 μm). (F) Expression of costimulatory molecules (CD80/CD86) in DC2.4 in different groups determined by flow cytometry. Typical inflammatory cytokines IFN-γ (G), TNF-α (H), IL-6 (I), and IL-10 (J) secreted by DC2.4 cells after different treatments (*P < 0.05, **P < 0.01, ***P < 0.001). I: Control; II: Model; III: RAPA; IV: HGPB; V: HGPBR; VI: HGPBRD; VII: MM@HGPBRD.

Atherosclerosis is a chronic inflammatory disease with increased intravascular ROS production throughout the disease process [38]. Overproduction of ROS and sustained oxidative stress can induce tissue and cellular damage, further initiating the inflammatory cycle and amplifying oxidative stress, which in turn promotes atherosclerosis progression. The excellent nanozyme activity of MM@HGPBRD in vitro stimulated us to further study their ROS clearance and anti-inflammatory effect in activated DC2.4 cells. Before that, we first assessed their cellular biocompatibility on DC2.4, RAW264.7, and 3T3 cells. As demonstrated in Fig. 4A to C, the cell survival rates were all above 80% after HGPBRD and MM@HGPBRD treatment with a concentration below 400 μg/ml. Additionally, under the same concentration, MM coating favored better cellular biocompatibility. Subsequently, we investigated whether MM@HGPBRD NPs could inhibit ROS generation in DC2.4 cells. LPS pretreated DC2.4 cells were used as model, and DC2.4 cells cultured in the medium were used as normal control. The generation of intracellular ROS was checked by the fluorescent probe DCFH-DA and examined by flow cytometry and inverted fluorescence microscope. As expected, compared with normal control cells, LPS-activated DC2.4 cells presented stronger fluorescence intensity, which was reduced by HGPB, HGPBR, HGPBRD, and MM@HGPBRD, showing the ROS-scavenging ability of our fabricated NPs in stimulated DC2.4 cells (Fig. 4D and E and Fig. S3). Furthermore, the presence of RAPA showed negligible influence on fluorescence intensity, illustrating that such ROS scavenging was mainly ascribed to the nanozyme ability of HGPB and aptamer targeting further enhanced this ability.

Clinical studies have found that a massive amount of mature DCs are detected in the plaque endothelium of atherosclerosis patients, suggesting that DC mature degree serves as a crucial status in the advancement of atherosclerosis [18]. RAPA can produce an immature phenotype on DCs, with a low expression of costimulatory molecules (CD80 and CD86) and a reduced generation of pro-inflammatory cytokines [24]. Therefore, we explored the ability of RAPA, HGPB, HGPBR, HGPBRD, and MM@HGPBRD to modulate costimulatory molecule expression in response to LPS inflammatory-provoked DC2.4 cells. The expressions of CD80 and CD86 in DC2.4 cells were significantly enhanced with LPS pretreatment, which were decreased in the presence of RAPA, HGPBR, HGPBRD, and MM@HGPBRD with varying degree, and the best inhibition effect was observed in the MM@HGPBRD group (Fig. 4F and Fig. S4). In the absence of RAPA, HGPB had less effect on the expression of DC2.4 cell surface molecules (CD80, CD86). We further investigated whether the above groups attenuated the inflammatory response of DC2.4 cells by ELISA method. Compared with HGPBR or free RAPA, MM@HGPBRD could significantly inhibit the secretion of pro-inflammatory cytokines such as IL-6, TNF-α, and IFN-γ and augment the generation of the anti-inflammatory cytokine IL-10 by LPS-stimulated DC2.4 cells (Fig. 4G to J). These results indicated that MM coating and DC-SIGN aptamer coupling might favor the intracellular delivery of MM@HGPBRD and significantly enhanced RAPA-based inhibition of DC pro-inflammatory pathways.

### Targeting efficacy of MM@HGPBRD in vitro

**Fig. 5.**
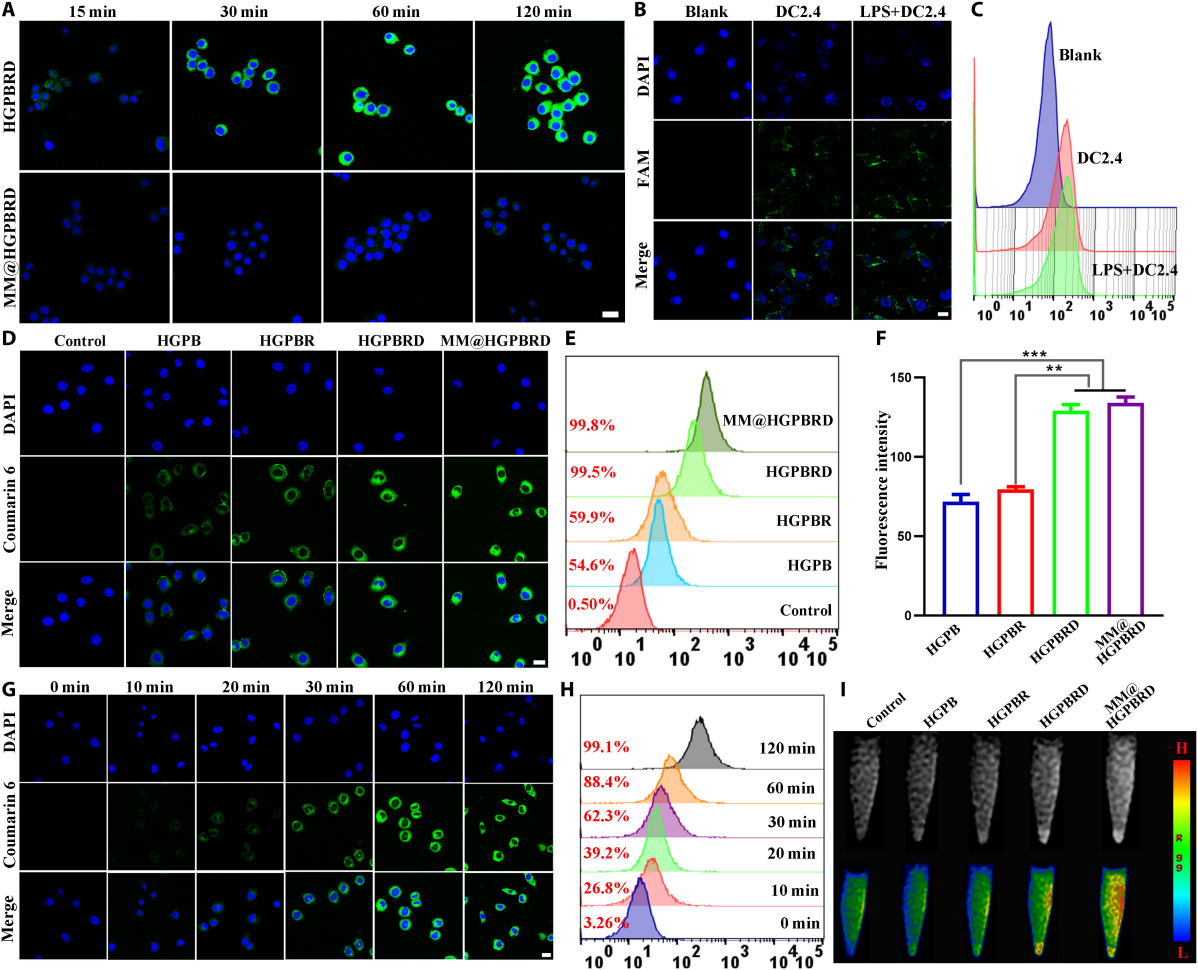
Targeting efficacy of MM@HGPBRD in vitro. (A) Confocal images of macrophages incubated with HGPBRD and MM@HGPBRD for different times (scale bar, 20 μm). The CLSM images (B) and flow cytometry analysis (C) of FAM-modified DC-SIGN aptamer binding with DC2.4 and LPS pretreated DC2.4 (scale bar, 20 μm). (D) The CLSM images of LPS-activated DC2.4 coincubated with different NPs [nuclei (DAPI, blue), NPs (C6, green); scale bar, 20 μm]. (E) Flow curves of different NPs taken up by LPS-activated DC2.4. (F) Semiquantitative analysis of fluorescence intensity in (D). (G) Confocal images of LPS-activated DC2.4 incubated with MM@HGPBRD for different times (scale bar, 20 μm). (H) Flow diagram of MM@HGPBRD NPs taken up by LPS-activated DC2.4 at different time points. (I) MR T1WI images and pseudocolor images of LPS-activated DC2.4 cells treated with HGPB, HGPBR, HGPBRD, and MM@HGPBRD (**P < 0.01, ***P < 0.001).

The above results indicated that the targeted delivery of drug might enhance the related effect such as the suppression of DC mature and the reduction of pro-inflammatory cytokines. Thus, we further confirmed such targeted ability of MM@HGPBRD to activated DC2.4 cells with fluorescence and MRI. First, the immune evasion was confirmed by CLSM. C6-loaded MM@HGPBRD and HGPBRD NPs were incubated with macrophages, and the results showed that more and more HGPBRD NPs were taken up by macrophages over time, while green fluorescence emission was barely noticeable in MM@HGPBRD-treated macrophages (Fig. 5A). The result indicated that MM@HGPBRD could evade macrophage phagocytosis with the help of specific proteins on cell membrane, which was conducive to extend the blood circulation time and enhance the accumulated amount of MM@HGPBRD in lesions. DC-SIGN aptamer was reported to bind to DC-SIGN on DC2.4 cells, and we incubated carboxyfluorescein (FAM)-modified DC-SIGN aptamer with DC2.4 cells with or without LPS pretreatment. As shown in Fig. 5B and C, DC-SIGN aptamer could bind to DC2.4 cells well. Then, the targeting behaviors of different NPs to DC2.4 cells were studied by flow cytometry and CLSM. C6-labeled HGPB, HGPBR, HGPBRD, and MM@HGPBR were incubated with LPS-activated DC2.4 cells. The fluorescence imaging and flow cytometry test displayed that the existence of DC-SIGN aptamer and MM significantly enhanced the targeting of NPs to activated DC2.4 cells, and the strongest targeting ability was observed in the MM@HGPBRD group (Fig. 5D to F). Furthermore, such fluorescence emissions were time dependent (Fig. 5G and H and Fig. S5). On the other hand, the excellent MRI ability of MM@HGPBRD (*r*_1_ = 40.22 mM^−1^ s^−1^ Gd; Fig. S6) enabled their MRI to activated DC2.4 cells. As demonstrated in Fig. 5I and Fig. S7, analogical tendency was observed, showing the outstanding targeting ability of MM@HGPBRD NPs to activated DC2.4 cells.

In total, the above results indicated that MM coating and DC-SIGN aptamer modification dramatically strengthened the ingestion of NPs by DCs, and this increased uptake could be attributed to the inflammatory targeting of MM and the binding of DC-SIGN aptamer to DC-SIGN on the surface of activated DC2.4 cells. It demonstrated that cascade targeting favored an enhanced ability of the nanodrug delivery system to target DCs at atherosclerotic sites and facilitated the effective delivery of immunosuppressive drugs.

### Specific MRI of atherosclerosis in vivo

**Fig. 6.**
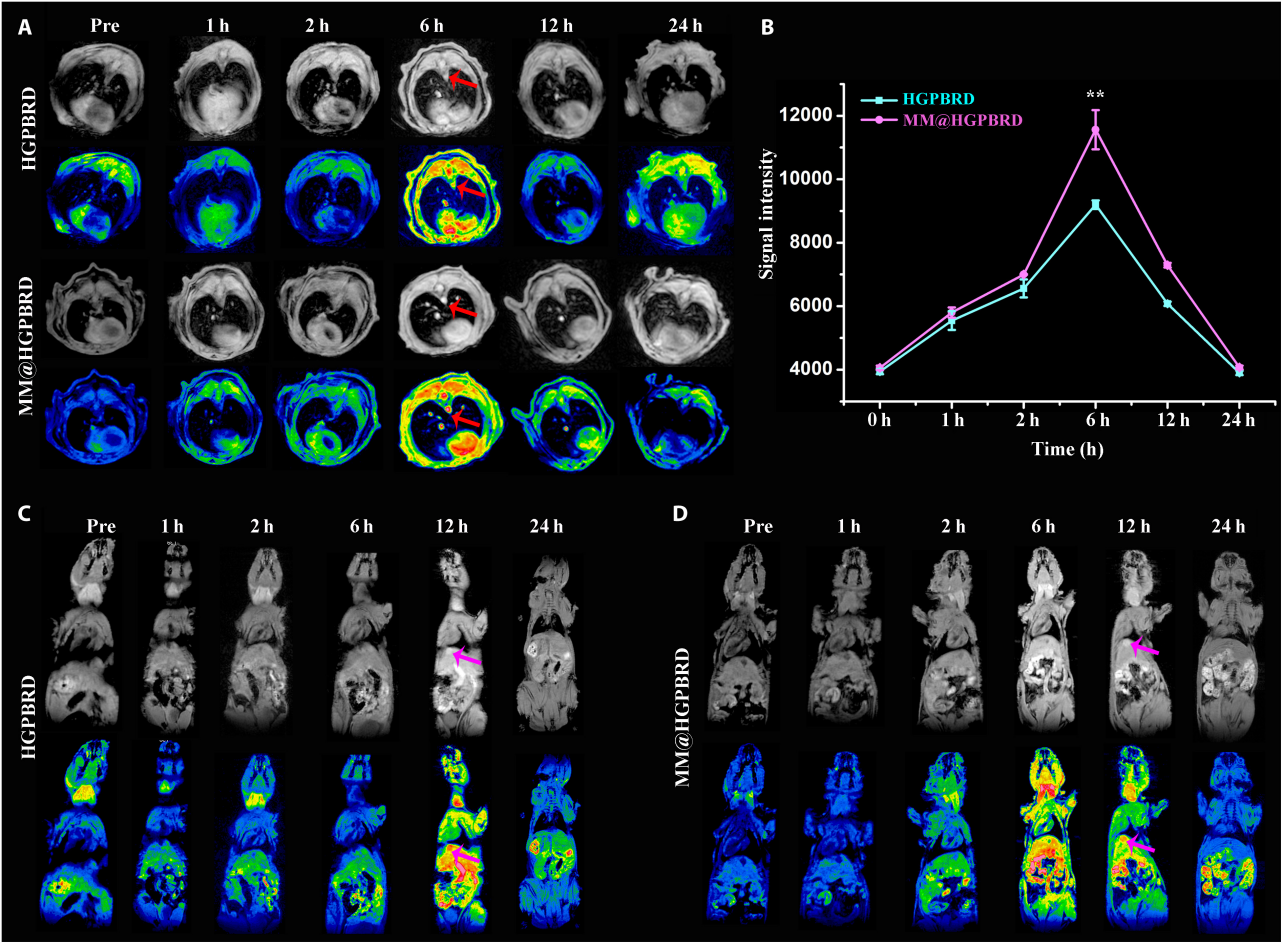
In vivo MRI. (A) Targeted MRI of aortic plaques (red arrow) in atherosclerosis model mice post-injection of MM@HGPBRD and HGPBRD via tail vein at 1, 2, 6, 12, and 24 h. (B) Quantitative analysis of magnetic resonance signal values of MM@HGPBRD and HGPBRD. MR images of atherosclerosis model mice after HGPBRD (C) and MM@HGPBRD (D) injection to show the metabolic process (pink arrow points to gallbladder) (**P < 0.01).

The excellent targeted fluorescence and MRI of MM@HGPBRD to activated DC2.4 cells in vitro encouraged us to carry out their in vivo specific MRI to DCs at the site of atherosclerotic plaques in ApoE^−/−^ mice. As shown in Fig. 6A, after injection of HGPBRD NPs or MM@HGPBRD NPs via tail vein, the T_1_ signals of aortic plaque gradually increased to maximum intensity within 6 h. Subsequently, the T_1_ signals diminished over time at 24 h. In addition, compared to the HGPBRD group, the T_1_ signal was stronger at 6 h in the MM@HGPBRD group (Fig. 6A and B), illustrating a better targeting ability to atherosclerotic plaques of MM@HGPBRD NPs. Such behavior was ascribed to the cascade targeting of MMs and DC-SIGN aptamer for their effectively escaping the clearance from the immune system and efficiently arriving to the atherosclerotic plaque sites in vivo. Exposure of MM@HGPBRD NPs to ROS environment of atherosclerosis could generate oxygen, which led to the rupture of the MM, and the exposure of DC-SIGN aptamer to characteristically target DCs at the atherosclerosis site. Furthermore, after injection of HGPBRD NPs and MM@HGPBRD NPs, a gradual increase in gallbladder MR signals was observed over time, with the signals reaching a maximum at 12 h and being completely cleared at 24 h, suggesting that they may be excreted through the hepatobiliary system (Fig. 6C and D). This was in agreement with the in vivo elimination of nanomaterials based on size effect [39].

### In vivo anti-atherosclerosis effect

**Fig. 7.**
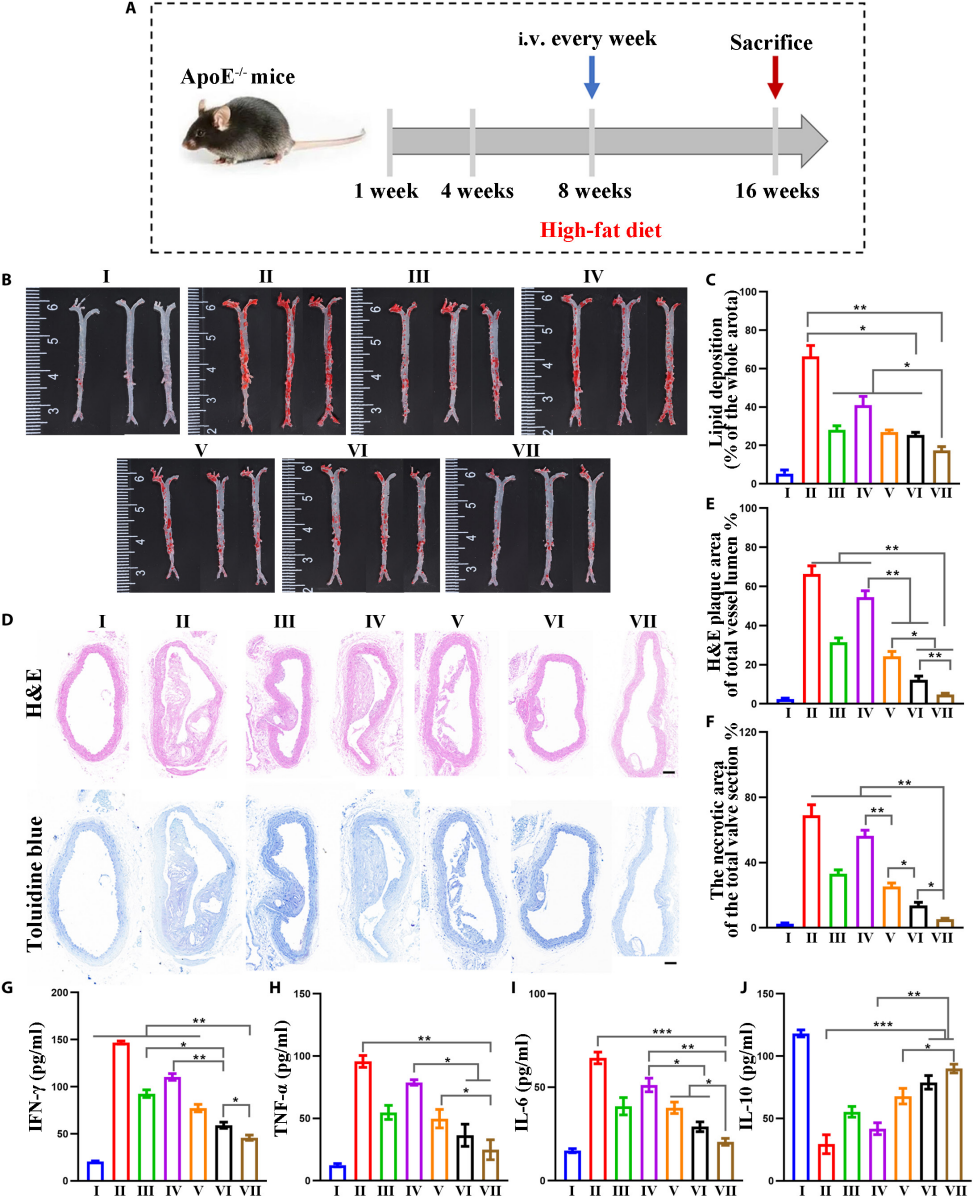
Evaluation of anti-atherosclerotic effect in vivo. (A) Schematic representation of therapy in atherosclerosis model mice. Oil red O staining (B) of aortic plaques and quantitative analysis (C) of plaque area in different groups. (D) H&E and toluidine blue (TB) staining of aortic plaques in each group (scale bar, 100 μm). Quantitative analysis of aortic plaques stained by H&E (E) and TB staining (F). Serum inflammatory secretion of IFN-γ (G), TNF-α (H), IL-6 (I), and IL-10 (J) (*P < 0.05, **P < 0.01, ***P < 0.001). I: Control; II: Model; III: RAPA; IV: HGPB; V: HGPBR; VI: HGPBRD; VII: MM@HGPBRD.

To study the efficacy of MM@HGPBRD NP treatment in vivo, ApoE^−/−^ mice were divided into 7 groups: control group (untreated mice with normal diet), model group (saline injection with high-fat diet), RAPA group, HGPB group, HGPBR group, HGPBRD group, and MM@HGPBRD group (Fig. 7A). After 2 months of high-fat diet, the latter 6 teams were post-injected with saline, RAPA (2.4 mg/kg), HGPB NPs (5 mg/kg), HGPBR NPs (5 mg/kg), HGPBRD NPs (5 mg/kg), and MM@HGPBRD NPs (5 mg/kg) via tail vein once a week for 8 weeks. For high-fat diet groups, there were no significant fluctuations in body weight before and after treatment in each group, indicating that MM@HGPBRD NP did not have a remarkable influence on the body weight of the mice (Fig. S8). After 8 weeks’ therapy, the aortas of each group were gathered and stained with oil red O. The bulk oil red O results showed that less area of plaque was presented in the control group and the largest area of plaque was presented in the model group, showing the successful fabrication of atherosclerosis model mice (Fig. 7B). After therapy, the aortic plaque area in HGPBRD NP and MM@HGPBRD NP groups was noticeably lower than that in the model group, and the smallest plaque area was found in the MM@HGPBRD NP group, illustrating that the MM@HGPBRD treatment (5 mg/kg for 8 weeks) significantly reduced ORO-stained atherosclerotic lesions. For further quantitative analysis, the plaque areas in the control group, model group, RAPA group, HGPB group, HGPBR group, HGPBRD group, and MM@HGPBRD group were determined to be 5.3 ± 1.9%, 66.3 ± 5.7%, 28.1 ±2.1%, 40.1 ± 4.6%, 26.8 ± 1.2%, 25.3 ± 1.5%, and 17.4 ± 2%, respectively (Fig. 7C). These consequences demonstrated that MM@HGPBRD NPs could successfully restrain the advancement of atherosclerosis.

Plaque area and plaque stability are the 2 predominant dimensions in assessing the efficacy of atherosclerosis treatment [40]. Therefore, atherosclerotic plaques in the aortic root after different treatments were histologically analyzed. Hematoxylin & eosin (H&E) staining showed that the aortic lesions in the model group, RAPA group, HGPB group, and HGPBR group were mainly made up of lipid-rich necrotic core. But in the HGPBRD group and MM@HGPBRD group, the area of necrotic zones was significantly lessened and the greatest reduction effect was observed in the MM@HGPBRD group (Fig. 7D and E). Toluidine blue staining can mirror the degree of plaque vulnerability by showing the size of necrotic nuclei within the plaque. Similarly, plaques in the model group were dominated by necrotic nuclei and cholesterol crystals, whereas plaque necrotic area was significantly reduced in the MM@HGPBRD group (Fig. 7D and F). This result also testified that MM@HGPBRD NPs could actively decrease plaque area and thus inhibit the development of atherosclerosis, which was in agreement with the outcome of macroscopic oil red O staining of the aorta.

Next, we determined the serum cytokines of the above groups after 8 weeks of treatment and found that compared with the model group, the pro-inflammatory cytokines IFN-γ, TNF-α, and IL-6 were reduced in the serum of the mice in all treated groups (Fig. 7G to I), whereas IL-10 was elevated (Fig. 7J). The suppression of serum pro-inflammatory cytokines and the elevation of anti-inflammatory cytokine were most obvious in the MM@HGPBRD group, illustrating the effective inflammation inhibition of MM@HGPBRD in atherosclerosis.

Taken together, these data suggested that MM@HGPBRD could enhance the therapeutic effects on atherosclerotic plaque inhibition and chronic inflammation. The MM immune evasion and inflammatory chemotaxis to reach the atherosclerotic site, the nanozyme activity of HGPB to improve the inflammatory microenvironment and expose DC-SIGN aptamer to realize cascade targeting to DCs in the site of atherosclerosis, and the inhibitory effect of RAPA collectively promoted the improvement of treatment efficiency.

### Inhibiting DC maturation and eliciting T_reg_ responses in vivo

**Fig. 8.**
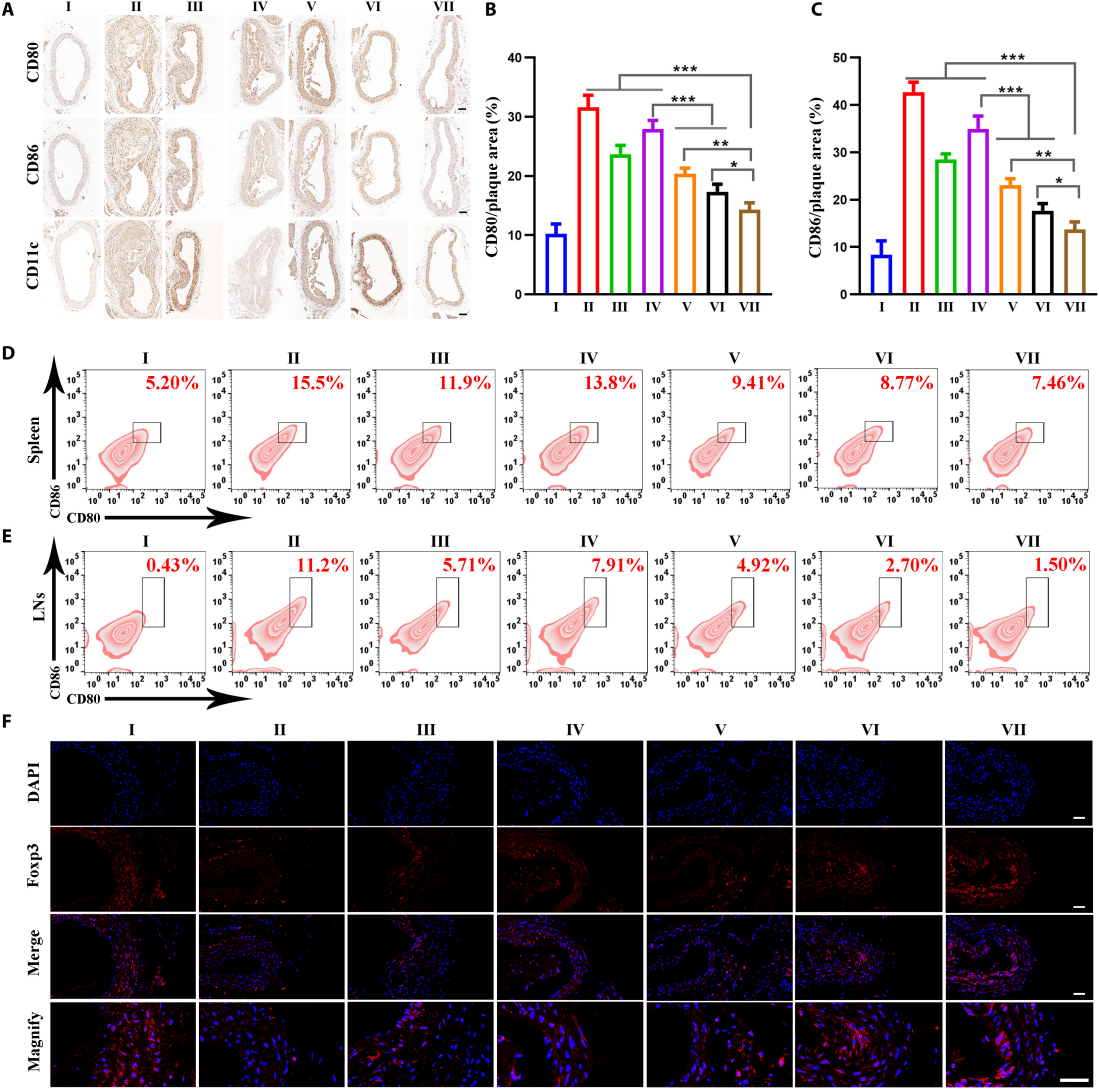
Evaluation of the in vivo immune response. (A) Immunohistochemical CD80, CD86, and CD11c staining of aortic plaques in each group (scale bar, 100 μm). Quantitative test of CD80 (B) and CD86 (C) in aortic plaques in each group. Cellular flow analysis of CD80/86 in spleen (D) and lymph nodes (E) in different groups. (F) Immunofluorescence assessment of Foxp3 expression in aortic plaques of different groups (scale bar, 50 μm) (*P < 0.05, **P < 0.01, ***P < 0.001). I: Control; II: Model; III: RAPA; IV: HGPB; V: HGPBR; VI: HGPBRD; VII: MM@HGPBRD.

DC-mediated antigen presentation has been known to occur in atherosclerotic lesions and peripheral lymphoid organs, where T cells migrate back into the lesion to manage the regional immune response [41]. Although mature DCs can activate naive T cells and initiate antigen-specific immune responses, immature DCs are known to tend to mediate tolerance [42]. In view of the fact that MM@HGPBRD NPs could inhibit DC maturation in vitro, we hypothesized that our DC-targeted NPs might induce atherosclerosis-protective T_reg_ responses. Therefore, we assessed the expression of the DC2.4 cell maturation markers CD80 and CD86 in lymphoid organs and atherosclerotic plaques after different treatment by immunohistochemistry and flow cytometry. In aortic plaques of ApoE^−/−^ mice, immunohistochemical and the corresponding quantitative analysis results showed that compared with the model group, no conspicuous reduction in the expression of CD80^+^CD86^+^ mature DCs in the CD11c^+^ population was seen in the HGPB group, which was memorably reduced in the RAPA group, HGPBR group, HGPBRD group, and MM@HGPBRD group, with the most significant reduction in the MM@HGPBRD group (Fig. 8A to C). It indicated that the inhibition of DC maturation at the atherosclerosis site by MM@HGPBRD mainly came from the role of RAPA. In the spleens of ApoE^−/−^ mice, the expression of CD80^+^CD86^+^ mature DCs in the CD11c^+^ population of the MM@HGPBRD group was dramatically lower than that of the model group, RAPA group, HGPB group, HGPBR group, and HGPBRD group, and was higher than that of the control group (Fig. 8D and Fig. S9). In the draining lymph nodes (DLNs) (Fig. 8E and Fig. S10), the trend of CD80^+^CD86^+^ mature DC expression in the CD11c^+^ population of the MM@HGPBRD group was consistent with that of the spleens. Taken together, the above results confirmed that MM@HGPBRD not only reached the plaque site to inhibit DC maturation but also affected the overall immune microenvironment changes to inhibit DC maturation in lymphoid organs.

Immunohistofluorescence was further employed to study the eliciting T_reg_ responses, and as shown in Fig. 8F and Figs. S11 and S12, compared with other groups, the levels of Foxp3^+^T_regs_ in atherosclerotic aortic plaques and spleen in the MM@HGPBRD group were significantly increased. MM@HGPBRD could inhibit DC maturation and promote the increase of anti-inflammatory T_regs_, which further regulated the systemic anti-inflammatory immune effect and eventually inhibited the progression of atherosclerosis.

In short, MM@HGPBRD not only effectively accumulated DCs in atherosclerotic plaques and inhibited the maturation of DCs but also scavenged ROS and reduced inflammation, which significantly enhanced the therapeutic efficacy of atherosclerosis treatment.

### Biocompatibility evaluation

**Fig. 9.**
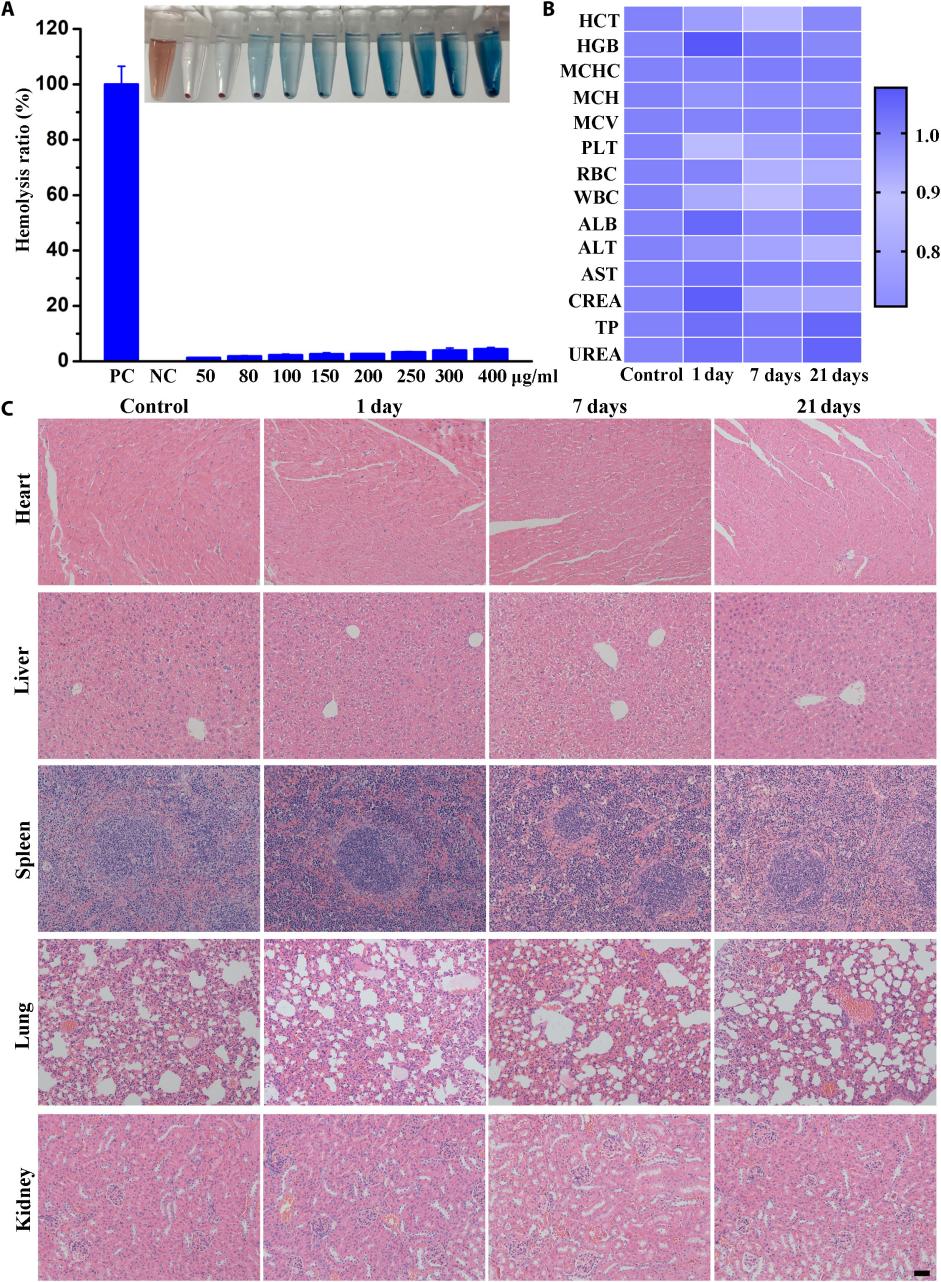
Biocompatibility evaluation. (A) Results of hemolysis experiments with different concentrations of MM@HGPBRD. (B) MM@HGPBRD hematological and blood biochemical indices in vivo. (C) H&E staining of major organ tissues after saline and MM@HGPBRD injection at 1, 7, and 21 d (scale bar, 100 μm).

We performed in vitro and in vivo safety assessments of MM@HGPBRD NPs, including hemolysis tests, measurement of serum markers of liver and renal function, and H&E staining analyses of principal organs. In the hemolysis test, MM@HGPBRD NPs did not induce significant hemolysis at any concentration, even at the highest concentration (400 μg/ml), which confirmed the blood safety of MM@HGPBRD NPs (Fig. 9A). Moreover, there were no significant movements in blood routine and blood biochemistry indices among different groups, and no obvious histological damage was observed in the MM@HGPBRD NP group (5 mg/kg), as attested by H&E staining of principal organs, confirming the in vivo safety of MM@HGPBRD NP treatment (5 mg/kg) (Fig. 9B and C).

## Conclusion

In conclusion, spontaneous induced cascade targeting biomimetic NPs, MM@HGPBRD, were successfully fabricated. The in vitro and in vivo consequences indicated their anti-atherosclerotic efficacy by inhibiting DC maturation, increasing T_reg_ infiltration, ROS scavenging, and anti-inflammation. The design of cascade targeting enhanced the therapy effect and MRI specificity to overcome the intricate microenvironment of atherosclerosis. This study offers a potential approach of the synergistic role of anti-inflammation and immune modulation for future therapy of atherosclerosis. In the future, the exploration of standardized methods for large-scale production and cryopreservation to innovate automated membrane harvesting technologies will further broaden the promising prospect of cell membrane-based biomimetic nanomaterials. Meanwhile, the construction of humanized disease models will favor accelerating clinic al translation.

## Data Availability

All data for the experiment are available on reasonable request.
