# Supplementary material for: Spontaneous Induced Cascade Targeting Biomimetic Nanoparticles to Inhibit Dendritic Cell Maturation for Ameliorating Atherosclerosis and Magnetic Resonance Imaging
Source: Biomater Res. 2025 May 9;29:0204. doi: 10.34133/bmr.0204 (PMC12062579; doi:10.34133/bmr.0204)
Supplement: Supplementary 1 — Table S1 Figs. S1 to S12 [file bmr.0204.f1.docx]

**Supplementary information**

**Spontaneous Induced Cascade targeting biomimetic nanoparticles to inhibit dendritic cell maturation for ameliorating atherosclerosis and MRI**

*Danyan Li*^1,2,#^, *Pengzhao Chang*^1,2,#^, *Shuang Bian*^1,2^, *Bangbang Li*^1,2^, *Yangang Zhu*^1,2^, *Yanchen Wang*^1,2^, *Pingfu Hou*^3,*^, *Jingjing Li*^1,2,*^

^1^School of Medical Imaging, Xuzhou Medical University, Xuzhou, 221004, People’s Republic of China;

^2^Department of Radiology, Affiliated Hospital of Xuzhou Medical University, Xuzhou, 221006, China

^3^Cancer Institute, Xuzhou Medical University, Xuzhou, Jiangsu, 221004, China

*Corresponding authors: Jingjing Li (Email: qingchao0124@163.com); Pingfu Hou (houpf612@xzhmu.edu.cn)

**Table S1 Drug loading and encapsulation rates of RAPA at different ratios**

| Mass ratio（HGPB:RAPA） | Drug loading efficiency | Encapsulation rate |
| --- | --- | --- |
| 10 : 1 | 2.4 % | 24.63 % |
| 5 : 1 | 5.4 % | 28.39 % |
| 2 : 1 | 14.5 % | 33.8 % |
| 1 : 1 | 27.4 % | 37.8 % |
| 1 : 2 | 48 % | 46.2 % |
| 1 : 5 | 72.1 % | 51.8 % |
| 1 : 8 | 81.7 % | 55.8 % |
| 1 : 10 | 83.1 % | 49.4 % |
| 1 : 12 | 82.1 % | 38.4 % |


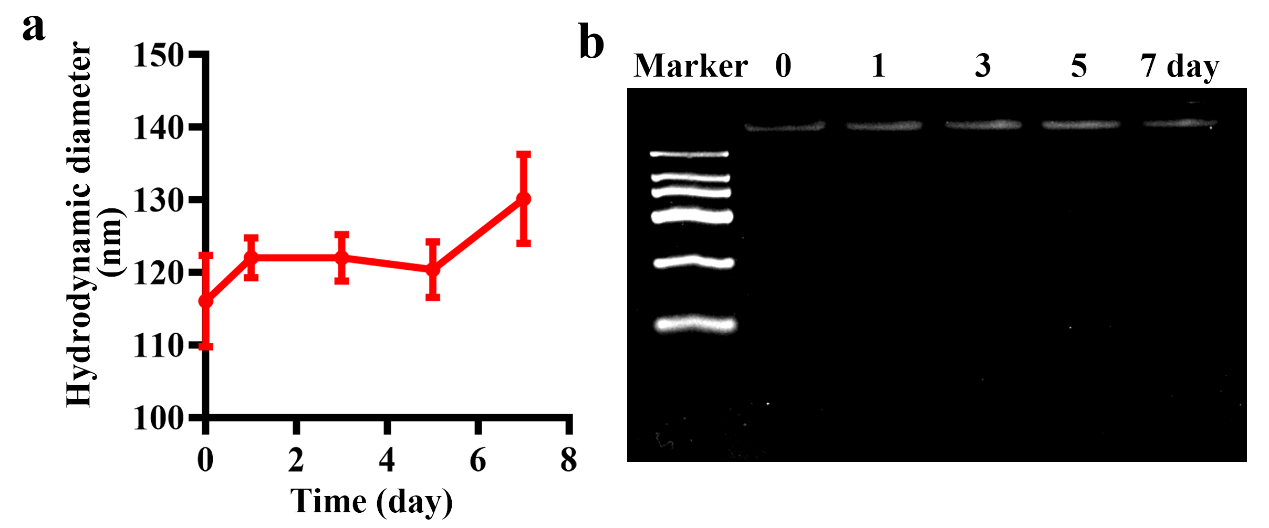


**Figure S1. Stability test of MM@HGPBRD.** (a) Hydrodynamic size change and (b) gel retardation assay during different storage time at 4 ℃.


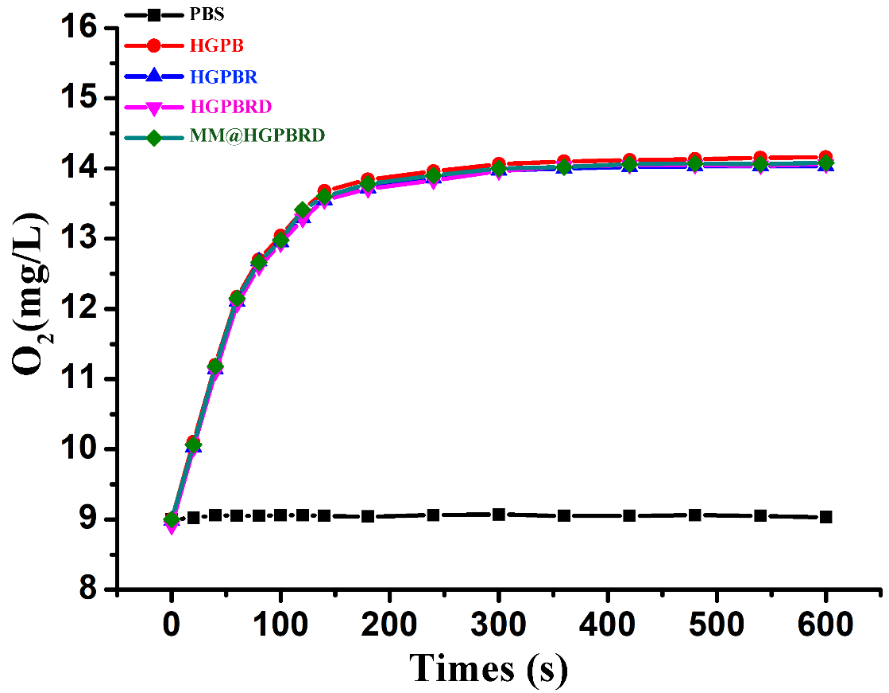


**Figure S2.** The amount of O_2_ produced by different nanomaterials under the same conditions.


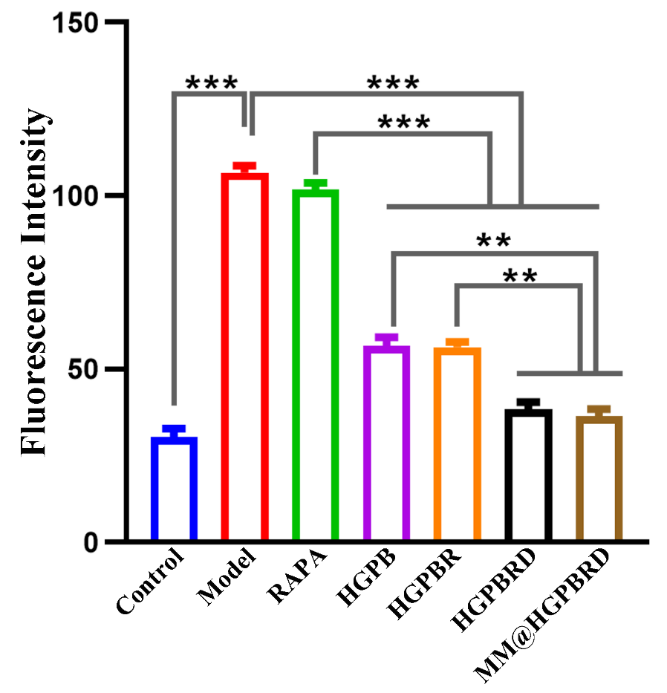


**Figure S3.** Quantitative analysis of ROS changes in DC2.4 cells in each group (**P < 0.01, ***P < 0.001).


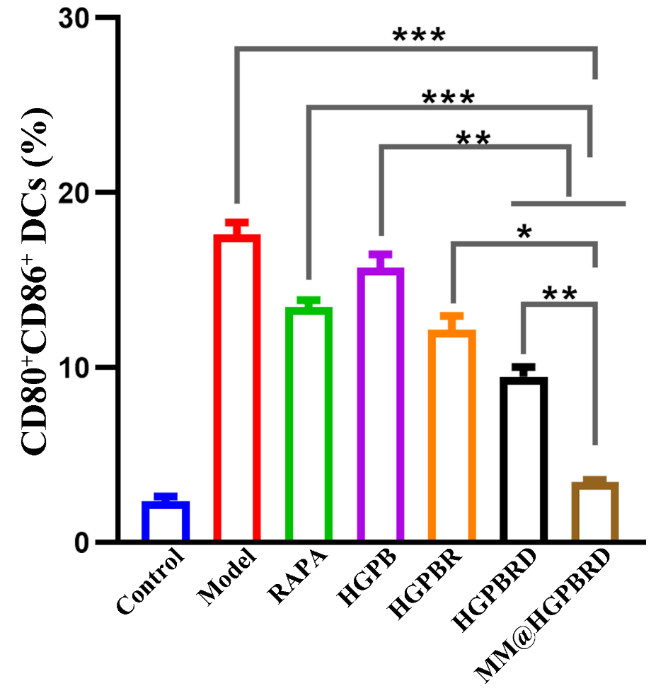


**Figure S4.** Quantification of CD80/CD86 expression levels in DC2.4 cells after different treatments (*P < 0.05, **P < 0.01, ***P < 0.001).


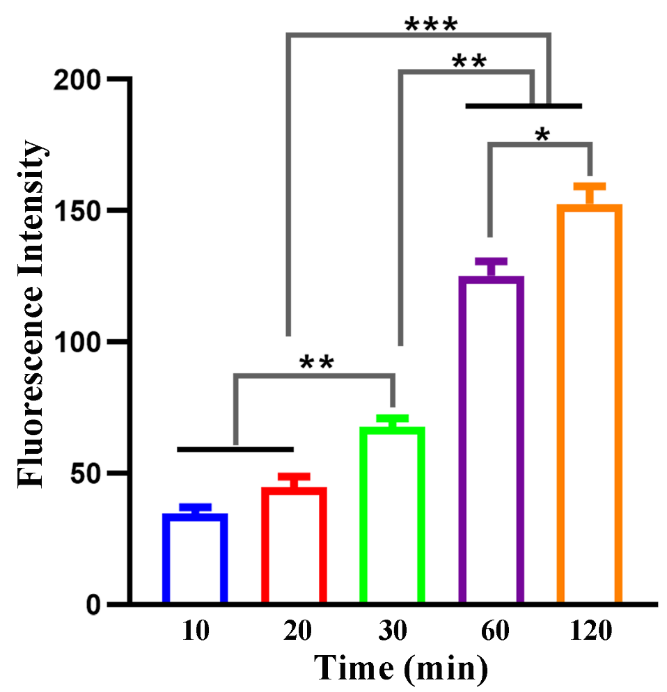


**Figure S5.** Quantitative analysis of the uptake fluorescence intensity of DC2.4 on MM@HGPBRD at different time points (*P < 0.05, **P < 0.01, ***P < 0.001).


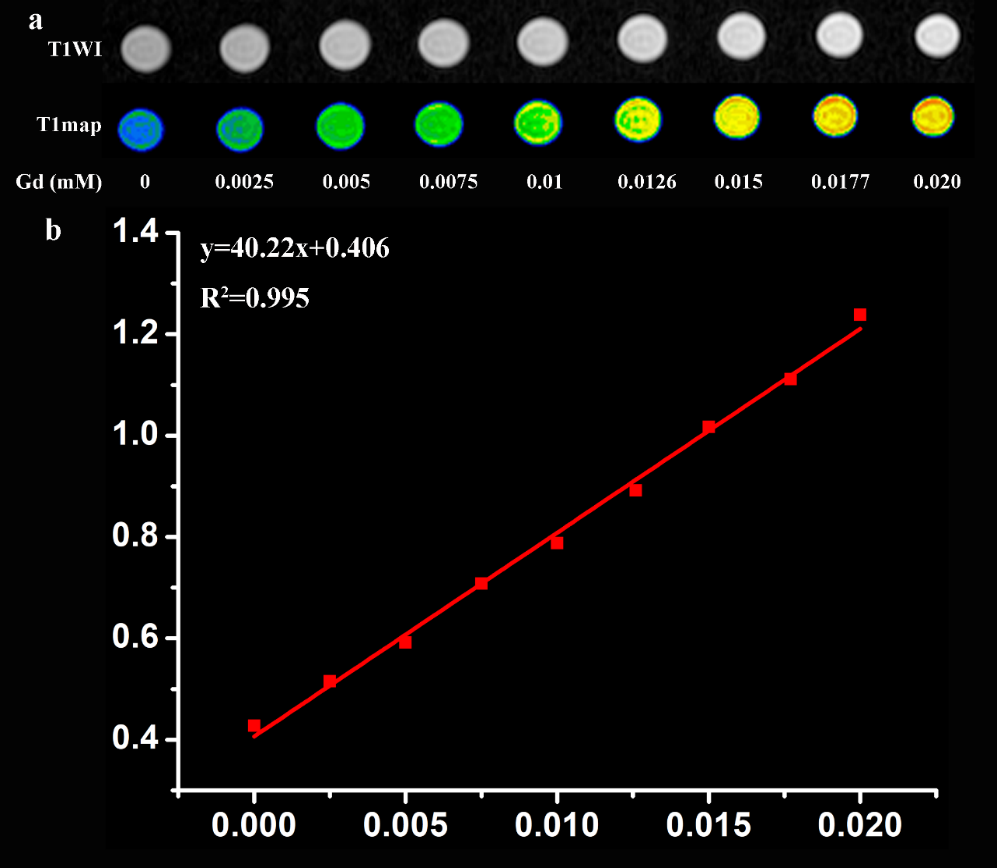


**Figure S6.** (a) T1WI and T1 mapping plots of MM@HGPBRD aqueous solutions with different concentrations; (b) T_1_ relaxivity curve of MM@HGPBRD.


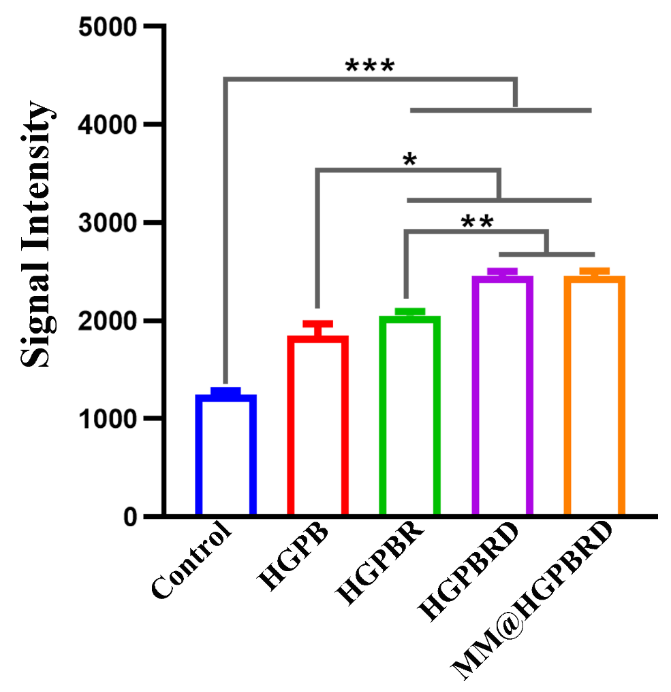


**Figure S7.** Quantitative analysis of magnetic resonance signal values for different groups (*P < 0.05, **P < 0.01, ***P < 0.001).


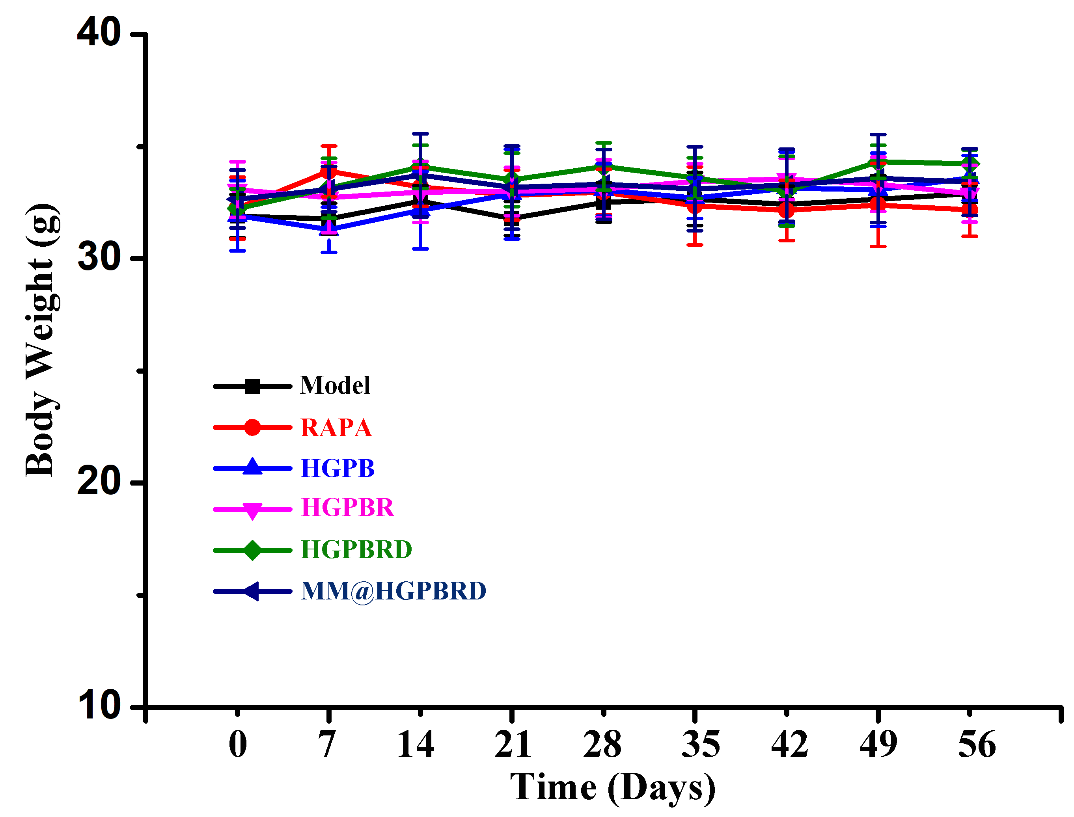


**Figure S8.** Changes in body weight of AS model mice in different treatment groups.


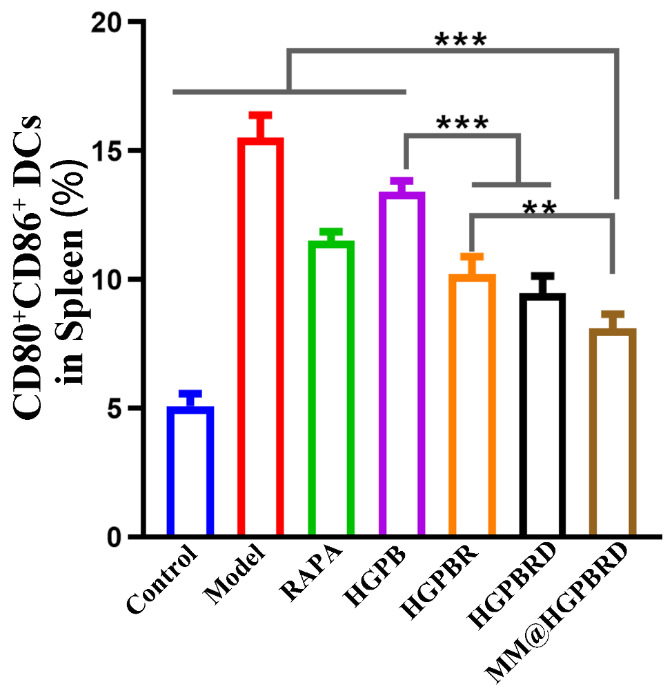


**Figure S9.** Quantitative analysis of CD80/86 in spleen of AS model mice in different treatment groups (**P < 0.01, ***P < 0.001).


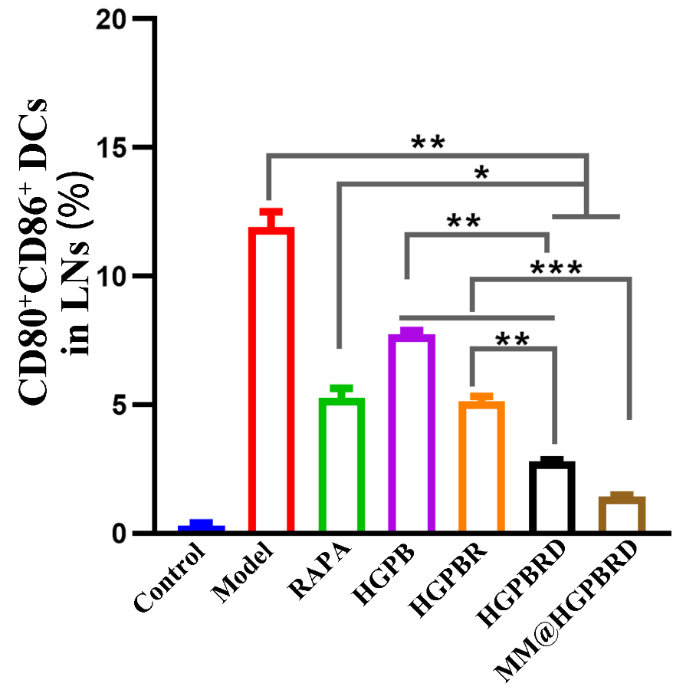


**Figure S10.** Quantitative analysis of CD80/86 in lymph nodes of AS model mice in different treatment groups (*P < 0.05, **P < 0.01, ***P < 0.001).


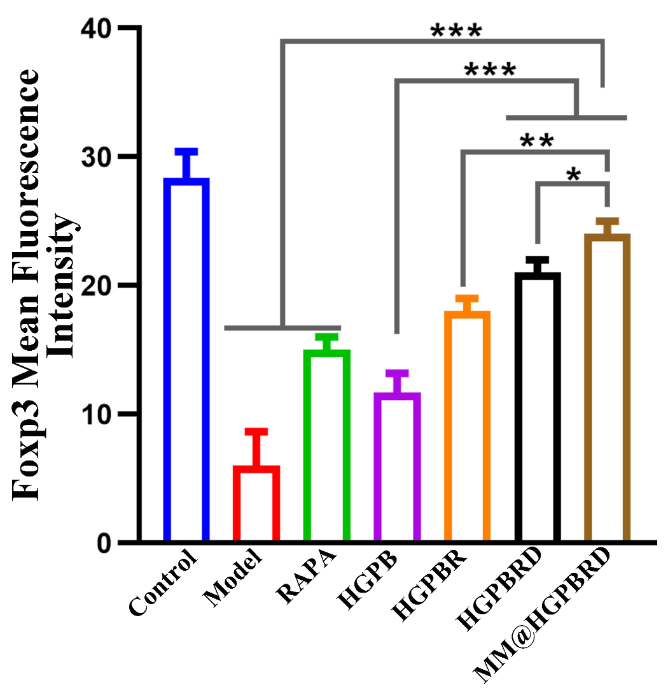


**Figure S11.** Quantitative measurement of Foxp3 expression in aortic plaques of different treatment groups (*P < 0.05, **P < 0.01, ***P < 0.001).


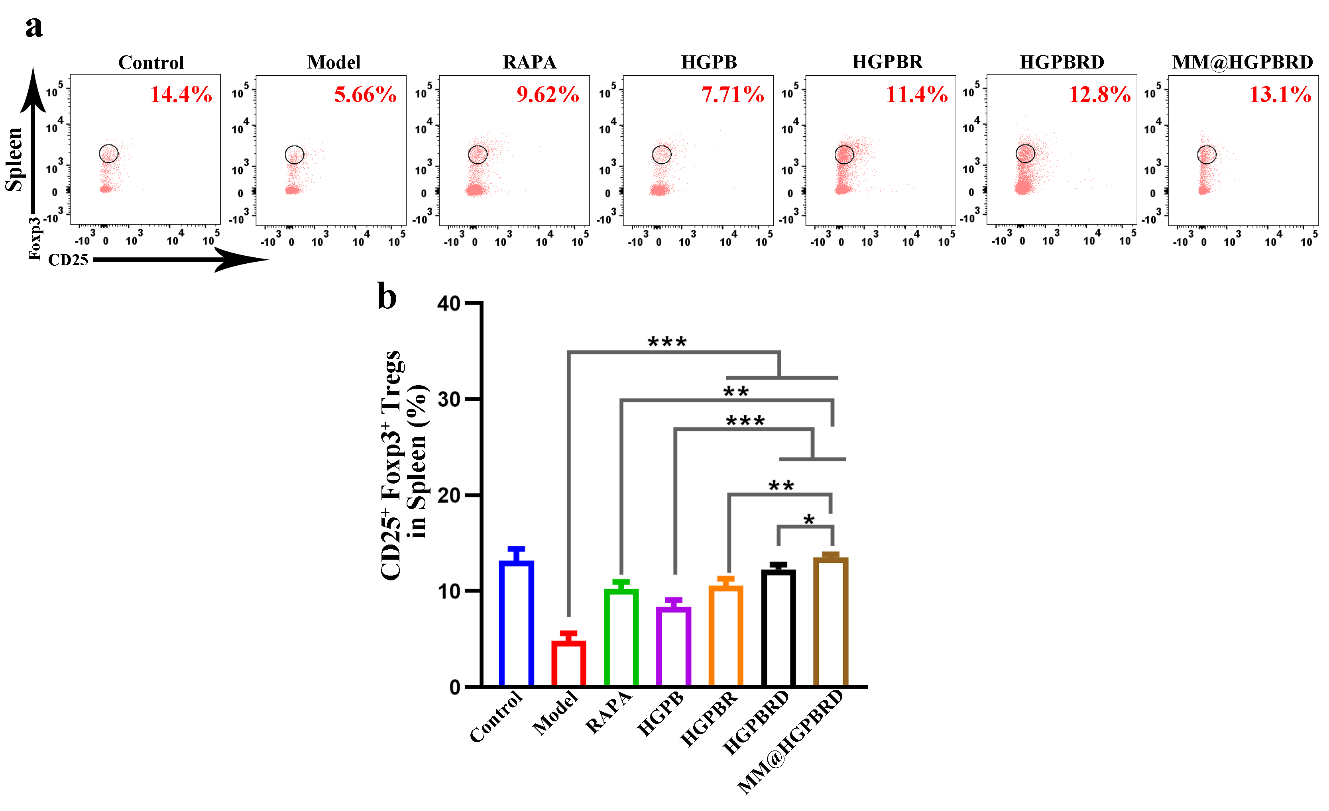


**Figure S12.** (a) Cell flow analysis of Foxp3 expression in the spleen of mice in different treatment groups; (b) Quantitative assessment of Foxp3 expression in the spleen of mice in different treatment groups (*P < 0.05, **P < 0.01, ***P < 0.001).
